# Global Analysis of the HrpL Regulon in the Plant Pathogen *Pseudomonas syringae* pv. *tomato* DC3000 Reveals New Regulon Members with Diverse Functions

**DOI:** 10.1371/journal.pone.0106115

**Published:** 2014-08-29

**Authors:** Hanh N. Lam, Suma Chakravarthy, Hai-Lei Wei, HoangChuong BuiNguyen, Paul V. Stodghill, Alan Collmer, Bryan M. Swingle, Samuel W. Cartinhour

**Affiliations:** 1 Department of Plant Pathology and Plant-Microbe Biology, Cornell University, Ithaca, New York, United States of America; 2 United States Department of Agriculture-Agricultural Research Service, Ithaca, New York, United States of America; University of the West of England, United Kingdom

## Abstract

The type III secretion system (T3SS) is required for virulence in the gram-negative plant pathogen *Pseudomonas syringae* pv. *tomato* DC3000. The alternative sigma factor HrpL directly regulates expression of T3SS genes via a promoter sequence, often designated as the “*hrp* promoter.” Although the HrpL regulon has been extensively investigated in DC3000, it is not known whether additional regulon members remain to be found. To systematically search for HrpL-regulated genes, we used chromatin immunoprecipitation coupled with high-throughput sequencing (ChIP-Seq) and bulk mRNA sequencing (RNA-Seq) to identify HrpL-binding sites and likely *hrp* promoters. The analysis recovered 73 sites of interest, including 20 sites that represent new *hrp* promoters. The new promoters lie upstream of a diverse set of genes encoding potential regulators, enzymes and hypothetical proteins. PSPTO_5633 is the only new HrpL regulon member that is potentially an effector and is now designated HopBM1. Deletions in several other new regulon members, including PSPTO_5633, PSPTO_0371, PSPTO_2130, PSPTO_2691, PSPTO_2696, PSPTO_3331, and PSPTO_5240, in either DC3000 or Δ*hopQ1-1* backgrounds, do not affect the hypersensitive response or *in planta* growth of the resulting strains. Many new HrpL regulon members appear to be unrelated to the T3SS, and orthologs for some of these can be identified in numerous non-pathogenic bacteria. With the identification of 20 new *hrp* promoters, the list of HrpL regulon members is approaching saturation and most likely includes all DC3000 effectors.

## Introduction

*Pseudomonas syringae* pv. *tomato* DC3000 (DC3000), an important model pathogen in molecular plant pathology, causes bacterial speck disease in *Arabidopsis* [1], tomato [2] and *Nicotiana benthamiana* (DC3000 mutants lacking virulence determinant HopQ1-1) [3]. The ability of DC3000 to colonize plants and subdue multiple layers of plant defense is dependent on the type III secretion system (T3SS) [4]. The T3SS machinery is encoded by the hypersensitive response and pathogenicity (*hrp*) and *hrp* conserved (*hrc*) gene clusters [5]. Effector proteins, encoded by *hrp* outer protein genes (*hop*) [6] are translocated into the host cytoplasm via the T3SS to interact with host proteins and/or intervene with host signaling cascades and responses for the benefit of the pathogen [7]–[9]. However, if one or more effectors or its activities are recognized by the plant immune system (through resistance proteins or other mechanisms), the host hypersensitive response (HR), a localized plant cell death, is triggered and bacterial growth is limited [10]. Effectors are examples of avirulence (avr) genes, a diverse group whose products typically stimulate a strong host defense response.

Efforts to identify genes involved in virulence and pathogenicity were initiated well before the genome sequences of DC3000 and other *P. syringae* strains were available. Experimental approaches included screening cosmid libraries for gain-of-function avirulence phenotypes [11], [12], using partial sequencing to characterize gene clusters flanked by pathogenicity islands [13], and identifying proteins secreted by the T3SS [14]–[16]. Regulation of the T3SS was linked to the alternative sigma factor HrpL when a DNA sequence of length 32 bases upstream of *hrpZ* was recognized to support HrpL-dependent transcription [17]–[19]. More comprehensive analyses of the HrpL regulon were possible once the genome sequence was determined [20]. For example, a combination of promoter trapping and sequence analysis was used to identify functional *hrp* promoters associated with pathogenicity [21]. This work established a consensus sequence for the putative *hrp* promoter. Another high-throughput screen, also based on promoter trapping, identified 29 T3SS proteins in DC3000 [22]. Although the search for T3SS effectors was suggested to be near saturation in this screen, it was carried out using a HrpL overexpression system which might have been vulnerable to false positives. A microarray screen comparing WT DC3000 and a Δ*hrpL* mutant [23] generated the currently accepted list of HrpL regulon members and 54 annotated *hrp* promoters, and has been considered to be nearly complete or exhaustive [24], [25]. More recently, HrpL overexpression and RNA-Seq were used to analyze the HrpL regulon in DC3000 as well as five other *P. syringae* strains, resulting in the identification of 14 new potential regulon members in DC3000 [26].

Although multiple approaches have been used to characterize the DC3000 HrpL regulon, several factors suggest that additional members may yet be found. First, inventory strategies have emphasized the identification and functional characterization of effectors, and thus non-effector genes that are important to bacterial growth and survival may have been overlooked. An example of a gene in this class is PSPTO_0834 (alcohol dehydrogenase, zinc-containing protein) [27], which is regulated by HrpL and strongly influences bacterial virulence. Second, effector identification is challenging because of effector redundancy and the frequent failure of effector gene mutants to exhibit a virulence phenotype. Many effectors, moreover, show no similarity to known proteins. Third, the *in planta* growth of D28E, a T3SS^+^ DC3000 derivative from which the 18 clustered effectors and 10 additional effectors were deleted, is significantly reduced relative to a T3SS^–^ control strain [28], [29]. This suggests that D28E expresses as yet unidentified factors that are recognized by the plant, some of which may be HrpL regulon members. Fourth, it is not yet known whether divergent examples of the *hrp* promoter, such as the one upstream of *iaaL* [23] actually bind HrpL or promote HrpL-dependent transcription. Finally, newly available genomic technologies offer enhanced sensitivity for the detection of transcriptional activity and promoter identification [26], [30], [31]. Together, these factors indicate that reexamining the HrpL regulon in DC3000 for additional members would be fruitful.

Here, we use chromatin immunoprecipitation coupled with high-throughput sequencing (ChIP-Seq), and a modified RNA-Seq protocol (incorporating the capture of mRNA 5′-ends), to provide evidence for direct binding of HrpL at *hrp* promoters and their activation by HrpL. The experiments identified most known members of the regulon (representing 52 out of 54 previously annotated HrpL-dependent promoters [15], [16], [21]–[25], [32]), as well as 20 new *hrp* promoter candidates. HrpL binding was validated using ChIP followed by quantitative polymerase chain reaction analysis (ChIP-qPCR) and promoter activity was tested using transcriptional fusions and quantitative reverse transcription-PCR (qRT-PCR). Computational analyses were used to search for additional members of the HrpL regulon in DC3000 and to investigate the conservation of HrpL regulon members and potential *hrp* promoters within the available *Pseudomonadales* genomes. This analysis revealed that some new HrpL regulon members are widely represented in both pathogenic and non-pathogenic bacteria. Finally, we conducted a translocation assay to demonstrate that one new HrpL regulon member, PSPTO_5633 (designated HopBM1), is translocated into plant cells in a T3SS-dependent manner.

## Materials and Methods

### Bacterial strains and growth conditions

DC3000 and its derivatives were grown at 28°C in MG medium [33], Kings B (KB) medium [34], HMM (*hrp*-minimal medium) [35], or on KB, MG, and HMM media solidified with 1.5% (wt/vol) agar. *Escherichia coli* TOP10 (Invitrogen) was used as the host for sub-cloning and other plasmid manipulations. *E. coli* was grown at 37°C in Luria-Bertani (LB) [36] medium or LB medium solidified with 1.5% (wt/vol) agar. Rifampicin, spectinomycin, kanamycin, and tetracycline were used at 50 µg/ml, 50 µg/ml, 50 µg/ml and 10 µg/ml, respectively. Rifampicin was added to medium used for growth of DC3000 derivatives. Other antibiotics were added to maintain plasmids as indicated in Table 1.

**Table 1:** All strains used in this study.

Designation	Genotype and Relevant Features	Reference
*Plasmids*		
pK18mobsacB	Small mobilizable suicide vector, sucrose-sensitive (sacB)/Kan^R^	[78]
pHL1	Destination vector for transcriptional fusion using LR reaction,carrying *gfp*/Tet^R^ Cam^R^	This study
pBS181	p*hrpJ*::iucD expresses GUS underT3SS inducing conditions/Tet^R^ Kan^R^	This study
pCPP6424	pENTR/SD/D-TOPO::PSPTO_5633; Entry vector withthe coding region of PSPTO_5633 lacking the stop codon/Kan^R^	This study
pCPP5371	Cya Gateway destination vector/Gen^R^ Cam^R^	[43]
pCPP6413	pCPP5371::PSPTO_5633/Gen^R^	This study
pCPP5388	pCPP5371::AvrPto/Gen^R^	This study
pBS62	pENTR/D with DC3000 genomic coordinates1542594.1542860; contains *hrpJ* promoter/Kan^R^	This study
pBS180	*iucD* reporter promoter trap destination vector; Gatewaycassette cloned upstream of *iucD*/Tet^R^ Kan^R^ Cam^R^	This study
pBS59	*lux* operon reporter promoter trap destination vector;Gateway cassette cloned upstreamof *lux* operon/Tet^R^ Kan^R^ Cam^R^	[40]
pTGS	Source of *gfp mut2*/Tet^R^	[41]
pBS12	*gfp* reporter promoter trap vector/Tet^R^	This study
HLN068	pENTR/SD/D-TOPO::P_PSPTO_0871_; Entry vector containingcandidate *hrp* promoter upstream from noted gene/Kan^R^	This study
HLN069	pENTR/SD/D-TOPO::P_PSPTO_0816_; Entry vector containingcandidate *hrp* promoter upstream from noted gene/Kan^R^	This study
HLN100	pENTR/SD/D-TOPO::P_PSPTO_2130_; Entry vector containingcandidate *hrp* promoter upstream from noted gene/Kan^R^	This study
HLN101	pENTR/SD/D-TOPO::P_PSPTO_4750_; Entry vector containingcandidate *hrp* promoter upstream from noted gene/Kan^R^	This study
HLN102	pENTR/SD/D-TOPO::P_PSPTO_5053_; Entry vector containingcandidate *hrp* promoter upstream from noted gene/Kan^R^	This study
HLN103	pENTR/SD/D-TOPO::P_PSPTO_5618_; Entry vector containingcandidate *hrp* promoter upstream from noted gene/Kan^R^	This study
HLN105	pENTR/SD/D-TOPO::P_PSPTO_1843_; Entry vector containingcandidate *hrp* promoter upstream from noted gene/Kan^R^	This study
HLN106	pENTR/SD/D-TOPO::P_PSPTO_3481_; Entry vector containingcandidate *hrp* promoter upstream from noted gene/Kan^R^	This study
HLN107	pENTR/SD/D-TOPO::P_PSPTO_3721_; Entry vector containingcandidate *hrp* promoter upstream from noted gene/Kan^R^	This study
HLN108	pENTR/SD/D-TOPO::P_PSPTO_4340_; Entry vector containingcandidate *hrp* promoter upstream from noted gene/Kan^R^	This study
HLN206	pENTR/SD/D-TOPO::P_PSPTO_5633_; Entry vector containingcandidate *hrp* promoter upstream from noted gene/Kan^R^	This study
HLN207	pENTR/SD/D-TOPO::P_PSPTO_0371_; Entry vector containingcandidate *hrp* promoter upstream from noted gene/Kan^R^	This study
HLN208	pENTR/SD/D-TOPO::P_PSPTO_1645_; Entry vector containingcandidate *hrp* promoter upstream from noted gene/Kan^R^	This study
HLN210	pENTR/SD/D-TOPO::P_PSPTO_2691_; Entry vector containingcandidate *hrp* promoter upstream from noted gene/Kan^R^	This study
HLN212	pENTR/SD/D-TOPO::P_PSPTO_2696_; Entry vector containingcandidate *hrp* promoter upstream from noted gene/Kan^R^	This study
HLN214	pENTR/SD/D-TOPO::P_PSPTO_3331_; Entry vector containingcandidate *hrp* promoter upstream from noted gene/Kan^R^	This study
HLN215	pENTR/SD/D-TOPO::P_PSPTO_3948_9_; Entry vector containingcandidate *hrp* promoter upstream from noted gene/Kan^R^	This study
HLN216	pENTR/SD/D-TOPO::P_PSPTO_4699_; Entry vector containingcandidate *hrp* promoter upstream from noted gene/Kan^R^	This study
HLN217	pENTR/SD/D-TOPO::P_PSPTO_4721_; Entry vector containingcandidate *hrp* promoter upstream from noted gene/Kan^R^	This study
HLN218	pENTR/SD/D-TOPO::P_PSPTO_4955_; Entry vector containingcandidate *hrp* promoter upstream from noted gene/Kan^R^	This study
HLN250	pENTR/SD/D-TOPO::P_PSPTO_5240_; Entry vector containingcandidate *hrp* promoter upstream from noted gene/Kan^R^	This study
HLN072	pHL1::P_PSPTO_0871_::*gfp*; Expression vector containingcandidate *hrp* promoter fused to *gfp* reporter/Tet^R^	This study
HLN162	pHL1::P_PSPTO_2130_::*gfp*; Expression vector containingcandidate *hrp* promoter fused to *gfp* reporter/Tet^R^	This study
HLN163	pHL1::P_PSPTO_4750_::*gfp*; Expression vector containingcandidate *hrp* promoter fused to *gfp* reporter/Tet^R^	This study
HLN164	pHL1::P_PSPTO_5053_::*gfp*; Expression vector containingcandidate *hrp* promoter fused to *gfp* reporter/Tet^R^	This study
HLN165	pHL1::P_PSPTO_5618_::*gfp*; Expression vector containingcandidate *hrp* promoter fused to *gfp* reporter/Tet^R^	This study
HLN167	pHL1::P_PSPTO_1843_; Expression vector containingcandidate *hrp* promoter fused to *gfp* reporter/Tet^R^	This study
HLN168	pHL1::P_PSPTO_3481_; Expression vector containingcandidate *hrp* promoter fused to *gfp* reporter/Tet^R^	This study
HLN169	pHL1::P_PSPTO_3721_; Expression vector containingcandidate *hrp* promoter fused to *gfp* reporter/Tet^R^	This study
HLN170	pHL1::P_PSPTO_4340_; Expression vector containingcandidate *hrp* promoter fused to *gfp* reporter/Tet^R^	This study
HLN219	pHL1::P_PSPTO_5633_; Expression vector containingcandidate *hrp* promoter fused to *gfp* reporter/Tet^R^	This study
HLN220	pHL1::P_PSPTO_0371_; Expression vector containingcandidate *hrp* promoter fused to *gfp* reporter/Tet^R^	This study
HLN221	pHL1::P_PSPTO_1645_; Expression vector containingcandidate *hrp* promoter fused to *gfp* reporter/Tet^R^	This study
HLN223	pHL1::P_PSPTO_2691_; Expression vector containingcandidate *hrp* promoter fused to *gfp* reporter/Tet^R^	This study
HLN225	pHL1::P_PSPTO_2696_; Expression vector containingcandidate *hrp* promoter fused to *gfp* reporter/Tet^R^	This study
HLN227	pHL1::P_PSPTO_3331_; Expression vector containingcandidate *hrp* promoter fused to *gfp* reporter/Tet^R^	This study
HLN228	pHL1::P_PSPTO_3948_9_; Expression vector containingcandidate *hrp* promoter fused to *gfp* reporter/Tet^R^	This study
HLN229	pHL1::P_PSPTO_4699_; Expression vector containingcandidate *hrp* promoter fused to *gfp* reporter/Tet^R^	This study
HLN230	pHL1::P_PSPTO_4721_; Expression vector containingcandidate *hrp* promoter fused to *gfp* reporter/Tet^R^	This study
HLN231	pHL1::P_PSPTO_4955_; Expression vector containingcandidate *hrp* promoter fused to *gfp* reporter/Tet^R^	This study
HLN253	pHL1::P_PSPTO_0816_; Expression vector containingcandidate *hrp* promoter fused to *gfp* reporter/Tet^R^	This study
HLN254	pHL1::P_PSPTO_5240_; Expression vector containingcandidate *hrp* promoter fused to *gfp* reporter/Tet^R^	This study
*Escherichia coli*		
TOP10	Φ80*lacZΔM15 ΔlacX74*	Invitrogen
*Pseudomonas syringae* pv. tomato		
DC3000	DC3000/Rif^R^	[79]
CUCPB5460	DC3000 with *hopQ1-1* deleted (*ΔhopQ1-1*)/Rif^R^	[3]
UNL-134–1	DC3000 with *hrpL* deleted (Δ*hrpL*)/Rif^R^ Spc^R^	[23]
DC3000pBS181	DC3000 carrying p*hrpJ*::iucD; expresses GUSunder T3SS inducing conditions/Tet^R^ Kan^R^ Rif^R^	This study
*hrpL-* *FLAG*pBS181	HLN090 carrying p*hrpJ*::iucD; expresses GUSunder T3SS inducing conditions/Tet^R^ Kan^R^ Rif^R^	This study
HLN090	DC3000 with *hrpL* tagged byFLAG at its C-terminus (*hrpL-FLAG*)/Rif^R^	This study
HLN010	DC3000 with *PSPTO_5633* on chromosomedeleted; remaining another copy on plasmid B/Rif^R^	This study
HLN009	DC3000 with hopQ1-1 and *PSPTO_5633* onchromosome deleted/Rif^R^	This study
HLN330	DC3000 with *PSPTO_5633* on plasmidand chromosome deleted/Rif^R^	This study
HLN012	DC3000 with *PSPTO_0371* deleted/Rif^R^	This study
HLN011	DC3000 with *hopQ1-1* and *PSPTO_0371* deleted/Rif^R^	This study
HLN014	DC3000 with *PSPTO_2130* deleted/Rif^R^	This study
HLN013	DC3000 with *hopQ1-1* and *PSPTO_2130* deleted/Rif^R^	This study
HLN016	DC3000 with *PSPTO_2691* deleted/Rif^R^	This study
HLN015	DC3000 with *hopQ1-1* and *PSPTO_2691* deleted/Rif^R^	This study
HLN018	DC3000 with *PSPTO_2696* deleted/Rif^R^	This study
HLN017	DC3000 with *hopQ1-1* and *PSPTO_2696* deleted/Rif^R^	This study
HLN020	DC3000 with *PSPTO_3331* deleted/Rif^R^	This study
HLN019	DC3000 with *hopQ1-1* and *PSPTO_3331* deleted/Rif^R^	This study
HLN182	DC3000 with *PSPTO_5240* deleted/Rif^R^	This study
HLN183	DC3000 with *hopQ1-1* and *PSPTO_5240* deleted/Rif^R^	This study
HLN190	Δ*pvsA* carrying pHL1::P_PSPTO_2130_::*gfp*; *gfp* under control ofpromoter upstream of *PSPTO_2130*/Rif^R^ Tet^R^ Kan^R^	This study
HLN191	ΔpvsA carrying pHL1::P_PSPTO_4750_::*gfp*; *gfp* under control ofpromoter antisense of *PSPTO_4750*/Rif^R^ Tet^R^ Kan^R^	This study
HLN192	ΔpvsA carrying pHL1::P_PSPTO_5053_::*gfp*; *gfp* under control ofpromoter upstream of *PSPTO_5053*/Rif^R^ Tet^R^ Kan^R^	This study
HLN193	ΔpvsA carrying pHL1::P_PSPTO_5618_::*gfp*; *gfp* under control ofpromoter upstream of *PSPTO_5618*/Rif^R^ Tet^R^ Kan^R^	This study
HLN195	ΔpvsA carrying pHL1::P_PSPTO_1843_::*gfp*; *gfp* under control ofpromoter upstream of *PSPTO_1843*/Rif^R^ Tet^R^ Kan^R^	This study
HLN196	ΔpvsA carrying pHL1::P_PSPTO_3481_::*gfp*; *gfp* under control ofpromoter upstream of *PSPTO_3481*/Rif^R^ Tet^R^ Kan^R^	This study
HLN197	ΔpvsA carrying pHL1::P_PSPTO_3720_::*gfp*; *gfp* under control ofpromoter upstream of *PSPTO_3720*/Rif^R^ Tet^R^ Kan^R^	This study
HLN198	ΔpvsA carrying pHL1::P_PSPTO_4340_::*gfp*; *gfp* under control ofpromoter upstream of *PSPTO_4340*/Rif^R^ Tet^R^ Kan^R^	This study
HLN232	ΔpvsA carrying pHL1::P_PSPTO_5633_::*gfp*; *gfp* under control ofpromoter upstream of *PSPTO_5633*/Rif^R^ Tet^R^ Kan^R^	This study
HLN233	ΔpvsA carrying pHL1::P_PSPTO_0371_::*gfp*; *gfp* under control ofpromoter upstream of *PSPTO_0371*/Rif^R^ Tet^R^ Kan^R^	This study
HLN234	ΔpvsA carrying pHL1::P_PSPTO_1645_::*gfp*; *gfp* under control ofpromoter upstream of *PSPTO_1645*/Rif^R^ Tet^R^ Kan^R^	This study
HLN237	ΔpvsA carrying pHL1::P_PSPTO_2691_::*gfp*; *gfp* under control ofpromoter upstream of *PSPTO_2691*/Rif^R^ Tet^R^ Kan^R^	This study
HLN238	ΔpvsA carrying pHL1::P_PSPTO_2696_::*gfp*; *gfp* under control ofpromoter upstream of *PSPTO_2696*/Rif^R^ Tet^R^ Kan^R^	This study
HLN240	ΔpvsA carrying pHL1::P_PSPTO_3331_::*gfp*; *gfp* under control ofpromoter upstream of *PSPTO_3331*/Rif^R^ Tet^R^ Kan^R^	This study
HLN241	ΔpvsA carrying pHL1::P_PSPTO_3948_3949_::*gfp*; *gfp* under controlof a *hrp* promoter in the middle*PSPTO_3948* and *PSPTO_3949*/Rif^R^ Tet^R^ Kan^R^	This study
HLN242	ΔpvsA carrying pHL1::P_PSPTO_4699_::*gfp*; *gfp* under control ofpromoter upstream of *PSPTO_4699*/Rif^R^ Tet^R^ Kan^R^	This study
HLN243	ΔpvsA carrying pHL1::P_PSPTO_4721_::*gfp*; *gfp* under control ofpromoter upstream of *PSPTO_4721*/Rif^R^ Tet^R^ Kan^R^	This study
HLN244	ΔpvsA carrying pHL1::P_PSPTO_4955_::*gfp*; *gfp* under control ofpromoter upstream of *PSPTO_4955*/Rif^R^ Tet^R^ Kan^R^	This study
HLN263	ΔpvsA carrying pHL1::P_PSPTO_0816_::*gfp*; *gfp* under control of apromoter upstream of of *PSPTO_0816*/Rif^R^ Tet^R^ Kan^R^	This study
HLN264	ΔpvsA carrying pHL1::P_PSPTO_0871_::*gfp*; *gfp* under control of apromoter upstream of *PSPTO_0871*/Rif^R^ Tet^R^ Kan^R^	This study
HLN265	ΔpvsA carrying pHL1::P_PSPTO_5240_::*gfp*; *gfp* under control of apromoter upstream of *PSPTO_5240*/Rif^R^ Tet^R^ Kan^R^	This study
HLN245	ΔpvsA carrying pHL1::P_φ_::*gfp*;; empty plasmid asnegative control in *gfp* assay/Rif^R^ Tet^R^ Kan^R^	This study
PS167	DC3000 *ΔpvsA*/Rif^R^ Kan^R^	[42]
DC3000T3SS-	CUCPB5113, DC3000*ΔhrcQ-U*/Rif^R^ Spc^R^	[80]
DC3000T2SS-	DC3000*ΔgspD*/Spc^R^	[81]
HLN397	DC3000 T3SS- carrying PSPTO_5633-Cya fusion (pCPP6413)/Rif^R^ Gen^R^	This study
HLN078	DC3000 carrying PSPTO_5633-Cyafusion (pCPP6413)/Rif^R^ Gen^R^	This study
HLN396	DC3000 T2SS- carrying PSPTO_5633-Cya fusion (pCPP6413)/Rif^R^ Gen^R^	This study
HLN079	DC3000 carrying AvrPto-Cya fusion(pCPP5388)/Rif^R^ Gen^R^	This study
HLN074	DC3000 T3SS- carrying AvrPto-Cyafusion (pCPP5388)/Rif^R^ Gen^R^	This study

Routine bacterial growth and medium shift experiments from *hrp*-inhibiting (KB medium) to *hrp*-inducing (MG supplemented with ferric citrate) conditions were carried out as follows. Colonies of *hrpL-FLAG* (in which the *hrpL* gene at its native locus has been tagged with a C-terminal FLAG epitope; see below for construction) and *ΔhrpL* were obtained from KB plates that had been incubated for 48 hours. Cells were re-suspended and grown overnight in 250 ml KB at 28°C with shaking at 250 rpm. Cultures were pelleted by centrifugation and washed in MG. Washed cells were re-suspended in MG and inoculated into bioreactors (Infors-HT, Switzerland) containing 400 ml MG medium supplemented with ferric citrate (Sigma-Aldrich) to a final concentration of 50 µM as described previously [33]. Samples were collected for RNA-Seq (5 ml) and ChIP-Seq (100 ml) at 1.5 hours after the medium shift into MG. Samples for RNA-Seq were supplemented with two volumes of RNAprotect Bacteria (Qiagen) to stabilize RNAs and stored at –70°C before RNA extraction. Samples for ChIP-Seq were immediately cross-linked with 37% Formaldehyde (1% final concentration) for 20 minutes. The crosslinking reaction was quenched with 2.5 M Glycine (0.36 M final concentration). Cells were collected and washed twice with Tris-buffered saline (TBS). Washed pellets were stored at –70°C until further processing.

### Construction of plasmids and strains

Suicide vectors for gene deletions, single-crossover insertions or other purposes were introduced into DC3000 backgrounds using electroporation [37]. Deletions created using pK18mobsacB (lacking FLP recombination target (FRT) cassettes) were performed as described previously [38]. Plasmid insertions into the bacterial chromosome were selected by plating on KB medium with kanamycin. Plasmid integration was confirmed by PCR, antibiotic resistance and sequencing.

#### Construction of hrpL-FLAG

Regions flanking the PSPTO_1404 *(hrpL)* gene were amplified by primers oSWC04110/oSWC04112 and oSWC04114/oSWC04116 (see Table S1 for all primer sequences) from DC3000 genomic DNA, purified by gel electrophoresis and gel extraction (Qiagen), and joined by SOEing PCR [39]. The joined fragment was then digested with XbaI (all enzymes were obtained from New England Biolabs unless otherwise noted), ligated with XbaI digested pK18mobsacB, and then transformed into *E. coli* TOP10. To ensure that the resulting construct was free from unwanted mutations, it was sequenced using primers M13F, M13R, oSWC05110, oSWC05111, oSWC05112, and oSWC05113. The FLAG-tagged construct was introduced into DC3000 by electroporation to generate HLN090. Merodiploid intermediates were selected for growth on medium containing kanamycin. Recombinants that had eliminated pK18mobsacB plasmid sequences were identified by sucrose counter-selection. Sucrose-resistant, Kan-sensitive colonies were analyzed by Sanger sequencing (with primers oSWC04110, oSWC04112, oSWC04114, oSWC04116, oSWC05110, oSWC05111, oSWC05112, and oSWC05113) to confirm successful tagging.

#### Mutants constructed for this study

A uniform strategy was used to construct deletions in *PSPTO_5633, PSPTO_0371, PSPTO_2130, PSPTO_2691, PSPTO_2696, PSPTO_3331,* and *PSPTO_5240.* Regions flanking the gene of interest, designated flank A and flank B, were amplified from DC3000 genomic DNA by two primer pairs (see Table S1), purified by gel electrophoresis (Qiagen), and joined by SOEing PCR [39]. The joined fragment was then digested with XbaI (or BamHI for *PSPTO_2691*), and ligated with XbaI (or BamHI) digested pK18mobsacB. The resulting constructs were sequenced to confirm correct structure using primers M13F, M13R, and the two primers flanking the deletion region. The deletion constructs were introduced into DC3000, and *ΔhopQ1-1* by electroporation. Merodiploid intermediates were selected for growth on medium containing kanamycin. Recombinants that had eliminated pK18mobsacB plasmid sequences were identified by sucrose counter-selection. Sucrose-resistant, Kan-sensitive colonies were screened by PCR using two primers flanking the deletion region. Mutants were confirmed by Sanger sequencing using four to six primers covering the manipulated region (see Table 1 for all mutants).

#### Construction of PhrpJ::iucD reporter plasmid

The plasmid pBS181encodes a transcriptional fusion of the *hrpJ* promoter with the *iucD* reporter gene (*P_hrpJ_*::*iucD*). The pBS181 plasmid was constructed by Gateway LR recombination (Invitrogen) between the pBS62 entry vector and the pBS180 destination vector. The pBS180 destination vector was constructed by replacing the *lux* operon of pBS59 [40] with the *iucD* gene. The *iucD* open reading frame was PCR amplified from pENTR-Gus (Invitrogen) using oSWC750 and oSWC751, digested with HindIII and ligated with the 8.6 kb fragment of HindIII digested pBS59, generating pBS180. The *hrpJ* promoter region was PCR amplified from DC3000 genomic DNA using oSWC463 and oSWC464 and cloned in pENTR/D-topo (Invitrogen) to yield pBS62. The *hrpJ* promoter *iucD* fusion was then constructed by LR reactions between pBS62 and pBS180 to yield pBS181.

#### Construction of promoter fusions

The *gfpmut2* gene was amplified from pTGS [41] using oSWC47 and oSWC48 for PCR. These primers introduced EcoRI recognition sequences to the 5′ and 3′ ends of the PCR product. The 5′ primer (oSWC47) also introduced a stop codon (TGA) in each frame (three total) followed by a Shine Delgarno sequence, which precedes the *gfpmut2* start codon by 8 base pairs (bps). The *gfpmut2* PCR product was digested with EcoRI and ligated to similarly digested pUCP24, yielding pBS12. pHL1 was constructed to function as a destination vector compatible with Gateway cloning. The Gateway cassette from plasmid pBS46 [40] was digested with enzyme KpnI and was ligated to plasmid pBS12 digested using the same enzyme. The resulting pHL1 construct contains a Gateway cassette, the promoter-less reporter gene *gfpmut2*, and antibiotic resistance genes for tetracycline and kanamycin resistance. To test for the presence of *hrp* promoters, fragments of DNA (150 bps to 200 bps) containing candidate promoters were cloned from DC3000 genomic DNA using the Expand High Fidelity PCR system (Roche, Basal, Switzerland). The forward primers have four additional bases (CACC) for compatibility with TOPO cloning vectors. PCR fragments were cloned into pENTR/SD/D by directional TOPO cloning and were subsequently used to generate the *gfp* reporter constructs by LR reaction with the pHL1 destination vector (Table 1) using LR Clonase II enzyme mix (Invitrogen). All constructs were confirmed by sequencing. Plasmids were transformed by electroporation [37] into DC3000 Δ*pvsA,* which differs from WT DC3000 in that it cannot produce pyoverdine, a fluorescent siderophore that makes accurate measurement of GFP concentration difficult [42].

#### Construction of PSPTO_5633–Cya fusion

The T3SS dependent translocation reporter gene *adenylate cyclase* [43] was fused to the C terminus of PSPTO_5633 using Gateway cloning (PCPP6413). The plasmids used to generate pCPP6413, containing the PSPTO5633-Cya fusion, are described in Table 1. Subsequently pCPP6413 and the translocation reporter control pCPP5388 (AvrPto-Cya) were conjugated into different *Pto* DC3000 strains by tri-parental mating using the helper plasmid pRK2013.

### Western blot

Proteins were resolved in a precast 4–20% polyacrylamide gel (Bio-Rad) and transferred to a PVDF membrane (Millipore) by electrophoresis. The membrane was then blocked in 5% non-fat milk for 2 hours at room temperature, and incubated with anti-FLAG M2 antibody (Sigma-Aldrich) for 1 hour at room temperature with gentle shaking. The membrane was washed three times in TBST buffer (50 mM Tris, 150 mM NaCl, 0.05% Tween-20, pH 7.6) and incubated with Alkaline Phosphatase conjugated 2^0^Antibody (Millipore) for 1 hour at room temperature. Proteins were detected after adding BCIP/NBT substrate (Sigma Aldrich) at room temperature.

### Chromatin immunoprecipitation with exonuclease Treatment (ChIP-exo) paired with high-throughput sequencing (ChIP-Seq)

ChIP-exo and ChIP-Seq were performed as described [42], [44]. Briefly, bacterial cultures were harvested and cross-linked with 1% formaldehyde final concentration. After 20 minutes incubation at room temperature with slow shaking, glycine was added at 0.36 M final concentration to quench the cross linking reaction. Cells were collected by centrifugation at 4°C for 5 minutes at 5,000×*g* and washed twice in ice cold TBS. Washed pellets were stored at –70°C until processed. To lyse cells, 1 ml of CelLytic B (Sigma-Aldrich), supplemented with 10 µl LongLife Lysozyme (1,500 U, G-Biosciences) and 10 µl PMSF (0.1 M, Sigma-Aldrich), was thoroughly mixed with thawed pellets by vortexing. Cells were incubated at 37°C for 10–15 minutes, and then disrupted by sonication (6 repetitions, 30 seconds each, with 2 minutes cooling between each pulse). Continuous pulse power at 15% power was used to produce fragments of size around 300 bps. For each ChIP-Seq sample, 40 µl of ANTI-FLAG M2 Affinity Gel (Sigma-Aldrich) was pre-washed in cold TBS, added to the bacterial lysates, and incubated with gentle shaking at 4°C for 2 hours. Unbound DNA fragments were eluted using two washes with TBS. Note that *hrpL-FLAG* samples were prepared following the ChIP-exo protocol that includes specialized enzyme treatment and library preparation procedures (all steps described below), while the *ΔhrpL* samples were eluted, reverse cross-linked (below) and directly submitted for high-throughput sequencing without additional manipulation as detailed previously [45].

For ChIP-exo, resin-bound DNA from the *hrpL-FLAG* samples were treated using methods adopted from Rhee *et al.* [31], with two TBS washes following each step:

1. End polishing was accomplished using 4.5 U of T4 DNA polymerase, 200 µM dNTPs and 50 µg/ml BSA at 12°C for 30 minutes. This reaction generates blunt end DNA fragments.
2. P2 adaptor (manufactured by IDT; see Table S1 for sequence) was ligated to both ends of the sheared DNA fragments using 1600 U of T4 DNA ligase and 200 ng adaptor at 25°C for 60 minutes. The P2 adaptor has only one blunt end that is available for ligation in this reaction.
3. Nick repair was accomplished using 20 U of Φ29 polymerase with 10 mM dNTPs and BSA at a final concentration of 100 ug/ml at 30°C for 20 minutes. This step generates double stranded DNA fragments without nicks.
4. The sample was digested with 10 U of λ exonuclease at 37°C for 30 minutes. This enzyme preferentially degrades double-stranded DNA from the 5′-end but is unable to degrade DNA regions protected by cross-linked protein. Unprotected double-stranded DNA is degraded in this step, yielding single-stranded DNA.
5. The sample was digested using 30 U of RecJ_f_ exonuclease at 37°C for 30 minutes. This enzyme is a single-stranded DNA specific exonuclease that digests DNA in the 5′ → 3′ direction. Unprotected single-stranded DNA from step 4 is largely destroyed by this step.

Resin-bound DNAs were separated from residual reaction buffers by centrifugation through Corning Costar spin-X centrifuge tube filters (Sigma-Aldrich), followed by two washes in ice cold TBS. DNA-protein complexes were eluted from resin using FLAG peptide (Sigma-Aldrich) at a final concentration of 150 ng/µl in 100 µl TBS, with slow shaking for 30 minutes. Supernatants were collected by centrifugation through the spin-X columns and saved as immunoprecipitated (IP) samples. To reverse formaldehyde crosslinking, IP samples were pre-incubated with 90 µl ChIP elution buffer (50 mM Tris-HCl pH 7.5, 10 mM EDTA, 1% SDS) and 10 µl of protease (Sigma-Aldrich) (40 mg/ml in TBS) for 2 hours at 42°C, followed by 6 hours at 65°C. Qiagen PCR-purification spin columns were used to purify DNAs.

To prepare ChIP-exo libraries for high-throughput sequencing, samples were incubated at 95°C for 5 minutes to denature double stranded DNAs. Primer P2 (5 pmol, see table S1) was added, allowed to anneal for two minutes at 30°C, and extended using 10 U of phi29 polymerase at 30°C for 20 minutes. The polymerase was inactivated by incubation at 65°C for 10 minutes. Blunt-end ligation of the P1 adaptor (10 uM) was accomplished using T4 DNA ligase 1000 U at 25°C for 60 min, followed by incubation at 65°C for 10 minutes to inactivate the ligase. DNA was purified using Agencourt AMPure magnetic beads and amplified by PCR using DNA polymerase Phusion in 18 PCR cycles. Finally, samples were purified using 80 µl Agencourt AMPure magnetic beads (Beckman Coulter Genomics), following the manufacturer’s instructions, and eluted to a final volume of 30 µl.

### RNA isolation and preparation for RNA-Seq

Total RNA was prepared using an RNeasy Kit (Qiagen) following the manufacturer’s instructions, using the optional on-column DNaseI digestion. RNA was treated twice with DNase I (Ambion) to remove residual DNA and then cleaned and concentrated using RNA cleanup and concentrator-5 (Zymo Research). Integrity of the RNA was assessed using the Agilent Bioanalyzer (Cornell University Life Sciences Core Laboratory Center Microarray Facility, Cornell University).

#### Depletion of processed RNAs and ligation of tag

Ribo-Zero rRNA Removal Kit (Epicenter) was first used to remove ribosomal RNA (rRNA). RNAs were then treated with Terminator 5′-Phosphate-Dependent Exonuclease (Epicentre), as described previously [46], to digest RNAs terminated by a 5′-monophosphate group, leaving RNAs terminated by 5′-triphosphate or 5′-hydroxyl groups undigested. Tobacco acid pyrophosphatase (TAP, Epicentre Biotechnologies) was then used to convert terminal 5′-triphosphate moieties to 5′-monophosphate. The 3′-ends were blocked by treatment with NaIO_4_ to prevent circularization before ligation of an RNA oligonucleotide (5′- ACA UCC ACA UCC UAG UAC −3′; IDT custom RNA oligo) to RNA 5′-ends. The ligation reaction was incubated overnight at 16°C. Products were recovered using RNA cleanup and concentrator-5 (Zymo research) and eluted in 10 µl H_2_O.

#### Construction of cDNA libraries for 5′ mapping and RNAseq

Stranded RNA-Seq libraries were prepared using the ScriptSeq v2 RNA-Seq Library Preparation Kit (Epicentre) and 500 pg to 50 ng rRNA-depleted RNAs following the manufacturer’s protocol. Briefly, RNAs were fragmented and cDNA synthesis was carried out using random-sequence primers containing a tagging sequence at their 5′-ends. The 3′-ends were tagged using the Terminal-Tagging Oligo. Di-tagged cDNAs were purified using magnetic beads and subjected to a limited-cycle PCR as recommended (10–15 cycles). Samples were indexed for multiplexing using the appropriate primers. Libraries were purified using the AMPure XP system (Beckman Coulter) in 20 µl total volume. Library quality was assessed using the Agilent Bioanalyzer. Sequencing was performed on the Illumina HiSEQ2000 by the Cornell DNA Sequencing Core Facility.

### Alignment of sequence reads to the DC3000 genome sequence and profile generation

For RNA-Seq data, reads bearing the unique 18 nucleotide sequence (tag) at the 5′ end were first identified and separated from the set of all RNA-Seq reads. The tag was then removed from each read, leaving the nucleotide sequences derived from the original RNAs. Thereafter, the de-tagged RNA-Seq reads (5′ capture data), the untagged RNA-Seq reads and reads obtained from the ChIP-exo procedure (ChIP-Seq data) were handled in the same way. Quality scores for the sequence reads were accessed using FastQC [47]. The first 75 nucleotides of each read with quality score of 20 or above (99% of inferred base calls are accurate) were aligned to the reference chromosome of DC3000 (accession number: AE016853), plasmid A (accession number: AE016855) and plasmid B (accession number: AE016854) using SOAPalign/soap2 [48].

Reads that aligned perfectly to a single location were retained and all others were discarded. The “sinister profiles” are histograms representing the number of trimmed reads whose 5′-ends uniquely map to each position [30]. A profile has values for each of the two strands of the genome. Profiles were visualized using the Artemis genome viewer [49] as previously described [30]. Profiles for the main chromosome and plasmids are available in Supplemental data.

### Identification of regions Enriched by ChIP

Enriched regions (those overrepresented in the ChIP-Seq data set) were identified using Genetrack [50]. The sinister profiles were formatted to a GeneTrack compatible format using a custom Python script. The GeneTrack analysis merged signals in cases where 5 adjacent positions had aligned read counts of 1000 or more at each position. Signals were constrained to be at least 700 bps apart from each other. Finally, overlapping signals on opposite strands were combined to generate ChIP-Seq ‘peaks’ (candidate HrpL-binding sites). Any signal mapping to one strand only (i.e., without an accompanying signal on the other strand) was discarded.

The normalized number of sequence reads associated with each site enriched by ChIP-Seq was also computed. The Un-normalized ‘peak’ height is an average of reads on both chromosomal strands in a window of 30 bps centered at ChIP-Seq ‘peak’. The background height was average of reads on both chromosomal strands in 2 windows of 30 bps that are 400 bps upstream and downstream of the ChIP-Seq ‘peak’. This background height was used to normalize the corresponding un-normalized ‘peak’ height. The normalized value on *ΔhrpL* mutant was also computed to present enrichment attributable to unspecific binding.

### Motif detection using MEME

Sequences of length 40–50 bps (FASTA format) upstream from captured 5′ transcription start sites were used as input to MEME [51] with the following parameters:

-dna -mod anr –nmotifs 20 -minw 14 -maxw 35 -maxsize 150000

The MEME package also generates sequence logos for each sequence pattern detected (Figure S1).

### HrpL regulon orthologs and matches to *hrp* promoter motifs

We first constructed a profile hidden Markov model (HMM) [52] using confirmed *hrp* promoters as a training set (Table S2). DNA sequences for 1060 closed and draft *Pseudomonadales* genomes were obtained from NCBI (Table S3). Prodigal [53] was used to generate uniform gene predictions for all genomes. Next, BLASTP [54] was used with an e-value threshold of 1e-6 to compare DC3000 protein sequences to proteins encoded in other genomes, and the reciprocal best matches were retained as presumptive orthologs. DNA sequences upstream from each DC3000 HrpL regulon ortholog were then extracted, with sequence length adjusted to account for the distance between the DC3000 promoter and its closest downstream gene (100 bps to 1500 bps), except for PSPTO_4750 (antisense sequence was extracted) and PSPTO_4955 (sequence in the middle of the gene was extracted). The sampled sequences were scanned with the profile HMM. In each case, the best scoring motif match in each upstream sample was noted as a potential *hrp* promoter (Table S3). The same HMM was used to scan the DC3000 chromosome to determine if additional potential *hrp* promoters could be associated with weak ChIP-Seq signals (Table S4). Custom scripts were used as necessary to simplify intermediate steps in the analysis.

### qRT-PCR and qPCR

cDNA synthesis was accomplished using qScript cDNA Synthesis (Quanta, Biosciences) and random primers as part of the kit. qPCR steps were performed using iQ SYBR Green Supermix (Bio-Rad). Primer pairs (see supplemental data) were selected to amplify a region of approximately 100 bps (Beacon Designer). For evaluating enrichment of ChIP-Seq binding sites, the primers amplified within the putative enriched regions. Enrichment (fold change) was calculated for DNA recovered at each tested binding site in the immune-precipitated (IP) sample compared to that in the lysate sample. The housekeeping genes, *gyrA* and *gap1*, were the internal and negative controls, respectively, for all tested regions. To test for transcript abundance, regions downstream of mapped transcriptional start sites were amplified. Transcript levels for each region (including the negative control, *gap1*) were calculated relative to the level for the housekeeping gene *gyrA*.

### Plant virulence assays

*Solanum lycopersicum* or *Nicotiana benthamiana* plants were germinated and grown in a greenhouse with approximate 16/8 hr. light/dark cycles. Four to five week old tomato plants or two to three week old *N. benthamiana* plants were inoculated with a 3×10^4^ CFU/ml bacterial suspension using blunt syringe infiltration. Bacteria were recovered from plants by sampling leaf tissue at the site of infection using a #2 disk punch (3 disks, total area 0.589 cm^2^) at 2 days post infection (dpi), 4 dpi and 6 dpi. Leaf disks were homogenized by mechanical disruption in 700 µl of 10 mM MgCl_2_. Serial dilutions of the tissue homogenate were plated on KB agar supplemented with rifampicin and the number of colony forming units per milligram leaf tissue was calculated.

### Hypersensitive Response (HR) assays

Inoculation was performed as described above for virulence assays except the inoculum concentration was 3×10^7^ CFU/ml for DC3000 derivatives unless otherwise noted. HR was observed and documented by photography after 2 days.

### Cya translocation reporter assays

Translocation assays were performed as described previously [43]. DC3000 strains were grown overnight at 28°C on KB agar medium with appropriate antibiotics, resuspended in 10 mM MgCl_2_, and adjusted to an OD_600_ of 0.05 (∼5×10^7^ CFU/ml). Bacterial suspensions were inoculated into *N. benthamiana* leaves using a blunt tip 1 ml syringe and plants were placed on the lab bench. After 6 hours, 2 leaf discs per sample were excised using a 1 cm-diameter cork borer and frozen in liquid nitrogen along with 2 copper-coated beads (Copperhead BBS, Crosman Corporation). The leaf discs were finely ground by vigorous vortexing and resuspended in 300 µl of 0.1 M HCl. The leaf extract was centrifuged at 6500 rpm for 10 minutes, and the supernatant was transferred to a fresh tube. A 10-fold dilution was prepared in 0.1 M HCl and used for the Cya assay since it was observed that diluting the extract resulted in greater sensitivity. Total pmol cAMP in each sample was determined using the Direct cAMP ELISA kit (Enzo Life Sciences) following the manufacturer’s instructions. Three plants were inoculated in each experiment and the samples were analyzed in 2–5 independent experiments.

## Results

### Candidate *hrp* promoter regions identified by ChIP-Seq

**Figure 1:**
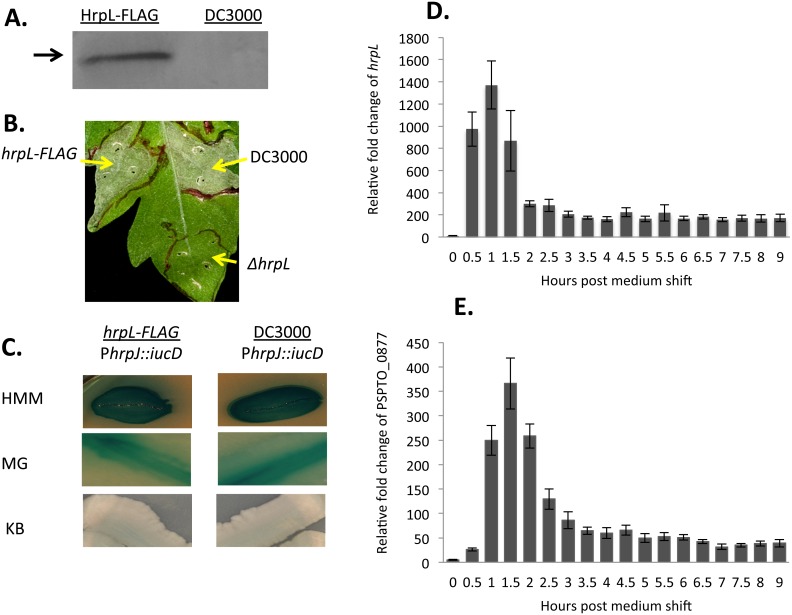
HrpL-FLAG is functional. (A) HrpL-FLAG is recognized by anti-FLAG antibody in a Western Blot. (B) *hrpL-FLAG* and WT DC3000 strains evoke the hypersensitive response while it is abolished in the *ΔhrpL* strain. Bacteria were infiltrated via a blunt syringe into three independent leaves at 3×10^8^ CFU/ml. Photos were taken after 3 days. Symptoms are identical among three replicates. (C) Expression of a plasmid-based (pBS181) β-glucuronidase (GUS) reporter driven by a *hrp* promoter (*hrpJ*) in *hrpL-FLAG* and WT DC3000 backgrounds. Three different media were used: HMM (*hrp*-minimal medium for highest induction of *hrp* promoters), MG (Mannitol-Glutamate medium for intermediate induction of *hrp* promoters but better bacterial growth), and KB (King’s B rich medium for repression of HrpL regulon expression). All plates contain appropriate antibiotics to maintain plasmids and X-Gluc, a substrate of GUS. (D-E) Relative fold change of transcript levels for *hrpL* and *hopQ1-1* (PSPTO_0877) after medium shift from KB to MG (supplemented with ferric iron at 50 µM final concentration) over 9 hours.

As a direct regulator of the HrpL regulon, the sigma factor HrpL (PSPTO_1404) is required for DC3000 virulence. To perform a global inventory of genomic sites likely to bind this sigma factor, we tagged HrpL with a FLAG epitope at the C-terminus by modifying the *hrpL* locus at its native position in DC3000 chromosome (Figure 1A) and performed a ChIP-Seq analysis. We first confirmed that cells bearing the tagged protein retained the ability to stimulate the hypersensitive response in a plant assay (Figure 1B) and established that HrpL-FLAG retained its ability to support transcription from a known HrpL-responsive promoter (Figure 1C). Results from a pilot experiment suggested that samples for ChIP-Seq should be harvested at 1.5 hours after a shift from non-inducing to *hrp*-inducing conditions, given the relatively high abundance of transcripts for *hrpL* and *hopQ1-1*, a confirmed effector, at this timepoint (Figures 1D and 1E).

Using the high-resolution ChIP-exo procedure described by Rhee *et al.* [44] we prepared samples for high-throughput sequencing. Table 2 summarizes the reads and mapping statistics of the two libraries using data generated by the Illumina Genome Analyzer. As a control, ChIP-Seq samples for the Δ*hrpL* strain were prepared using a conventional ChIP-Seq protocol [45] that omits the steps in ChIP-exo that destroy most of unbound DNA [44] (described in Materials and Methods). The read counts from this sample were compared to those from the *HrpL-FLAG* sample to determine levels attributable to non-specific binding.

**Table 2:** Read mapping statistics.

Sample	Sequence read type	*hrpL-FLAG*	*ΔhrpL*
ChIP-Seq	Total reads	45,479,930	22,170,715
	Reads that align uniquely to the chromosome	35,185,956	9,513,703
	Reads that align uniquely to plasmid B	2,699,119	571,096
	Reads that align uniquely to plasmid A	3,308,504	573,047
RNA-Seq	Total reads	36,272,771	44,500,185
	Reads containing 18-mer	356,915	3,351,960
	Untagged reads that alignuniquely to the chromosome	29,349,347	32,953,858
	Untagged reads that align uniquely to plasmid B	1,426,734	515,184
	Untagged reads that align uniquely to plasmid A	878,164	426,741
	Tagged reads that align uniquely to thechromosome	237,794	2,069,913
	Tagged reads that align uniquely to plasmid B	20,570	30,903
	Tagged reads that align uniquely to plasmid A	7,452	30,501

Using the Genetrack [50] analysis package, we identified 73 enriched regions in the DC3000 genome, of which 52 can be associated with one of the 54 annotated *hrp* promoters (Table S5), and 21 appear in regions without known *hrp* promoters (Table 3). The two annotated promoters that were negative with respect to enrichment were PSPTO_1370 (type III effector HopN1) and PSPTO_3489 (a sugar ABC transporter/ATP-binding protein). Visual inspection of these regions in the ChIP-Seq profile also showed no evidence of enrichment. Because PSPTO_5633 and PSPTO_B0003 have identical coding and upstream sequences, we counted them as a single enrichment instance, reducing the number of novel regions identified by ChIP-Seq to 20. Among these promoter candidates, only two are obviously associated with genes that encode proteins involved in the T3SS, namely PSPTO_4721 (type III chaperone ShcV) and PSPTO_5618 (pseudogene for type III effector HopAT1). The remaining candidates are upstream of genes encoding enzymes, hypothetical proteins or proteins with other functions.

**Table 3:** Data for new *hrp* promoters.

Operon	Function	*hrp* promoter coordinate	Evidence	qRT-PCR	Published data		
					Mucyn*et al.*	Promoters	Genes
371	iaaL indoleacetate-lysine ligase	406210.406238	HBIS	1151.0±177.6	+	FM	FCM_o_
871	macrolide effluxprotein, putative	939675.939703	HBIS	31.9±3.6	+		M_o_
1645	MarR familytranscriptional regulator	1802305.1802333	HBIS	(54.6±14.6)*		M	M
1843	aspartate kinase	2012108.2012136	HBIS	2.2±0.2	+		M_o_
2130	LuxR family DNA-binding response regulator	c(2304331.2304359)	HBIS	57.5±11.2	+	M	M_o_
2691	TerC familymembrane protein	2984435.2984463	HBIS	49.9±6.0	+		M
2696	mutT/nudixfamily protein	(2990249.2990277)	HBIS	29.2±5.0		M	M_o_
3331	protease inhibitor Inh	c(3768950.3768978)	HBIS	17.1±5.2			M_o_
3481	Hypothetical protein	3929005.3929032	HBS	(15.6±1.6)*			M_1_
3721	fabI enoyl-(acyl-carrier-protein) reductase	4199604.4199632	HBIS	(1.4±0.1)*		M	M_o_
39483949	Intergenic region	c(4457004.4457031)	HBI	(5.0±1.4)*			M_1_
4340	insecticidal toxinprotein, putative	c(4895201.4895228)	HBIS	(3.1±0.3)*			M_o_
4699	non-ribosomal peptidesynthetase, terminal	c(5328022.5328050)	HBIS	223.4±57.9		M	M_o_
4721	Type III chaperone ShcV	c(5346761.5346788)	HBIS	439.7±164.1	+		M_o_
4750	Hypothetical protein	5384480.5384507	HBIS	62.8±17.8			M_o_
4955	bifunctional thiosulfate	5616671.5616699	HBIS	13.7±3.6		M	M_o_
5053	Hypothetical protein	5751475.5751504	HBIS	(209.7±50.5)*		M	M_o_
5240	CDP-6-deoxy-delta-3,4-glucoseen reductase	c(5960164.5960192)	HBIS	8.6±1.0			M_o_
5618	Type III effectorpseudogene hopAT1	c(922925.922953)	HIS	99.9±7.2		M	M_1_
5633	conserved protein ofunknown function	15821.15849	HBIS	61.2±9.4			M_1_
B0003	(identical to PSPTO_5633)						

Operon: PSPTO identifier for gene immediately downstream from promoter.

Function: annotated function for operon-identifying gene.

Coordinate: DC3000 genome coordinate for the region bracketing the –35 and –10 regions of the promoter. “c” designates that the promoter is found on the complementary strand.

Evidence: Experimental evidence for *hrp* promoters from this study.

• H: *hrp* promoter motif found;

• B: Binding activity for HrpL (ChIP-qPCR) observed;

• I: Induction observed in promoter fusion (threshold = 2.4; 2×negative control);

• S: mRNA 5′-end captured (TSS) within 10 bps from the 3′ end of −10 promoter element. No threshold is applied. Absolute values for read counts appear in Table S5. TSSs from Filiatrault *et al*. [46] were also taken into consideration.

qRT-PCR: Transcript abundance for regions downstream from *hrp* promoters in DC3000 compared to that in a *ΔhrpL* strain. Values in brackets indicate that abundance was measured upstream from coding region. Values are average of one or two biological replicates with three technical replicates and standard deviation.

Published data:

• Mucyn *et al.*: ‘+’ indicates that the gene was classified as differentially expressed in Table 3 and Table S3 by Mucyn *et al.* [26].

• Promoters: symbols indicate whether *hrp* promoters were reported by Chang *et al.* [22] (C), Fouts *et al.* [21] (F), or by Ferreira *et al.* [23] (M).

• Genes: symbols indicate whether genes downstream from *hrp* promoters were tested and/or reported by Chang *et al.* [22] (C), Fouts *et al.* [21] (F), or by Ferreira *et al.* [23] (M). Genes that showed no differential expression, or which were not tested by Ferreira *et al.* [23] are designated (M_o_) and (M_1_), respectively.

No binding evidence for HrpL or upstream *hrp* promoter motifs were observed in association with 12 of the 14 genes proposed by Mucyn *et al.* [26] as putative novel HrpL regulon members. These are PSPTO_0829 (clpB protein), PSPTO_0851 (hypothetical protein), PSPTO_1371 (effector locus protein), PSPTO_2129 (sensory box histidine kinase/response regulator), PSPTO_2208 (heat shock protein HtpG), PSPTO_3148 (magnesium chelatase subunit ChII), PSPTO_4210 (ATP-dependent protease La), PSPTO_4332 (hypothetical protein), PSPTO_4376 (chaperonin, 60 kDa), PSPTO_4505 (dnaK protein), PSPTO_4716 (hypothetical protein), and PSPTO_4723 (hypothetical protein).

**Figure 2:**
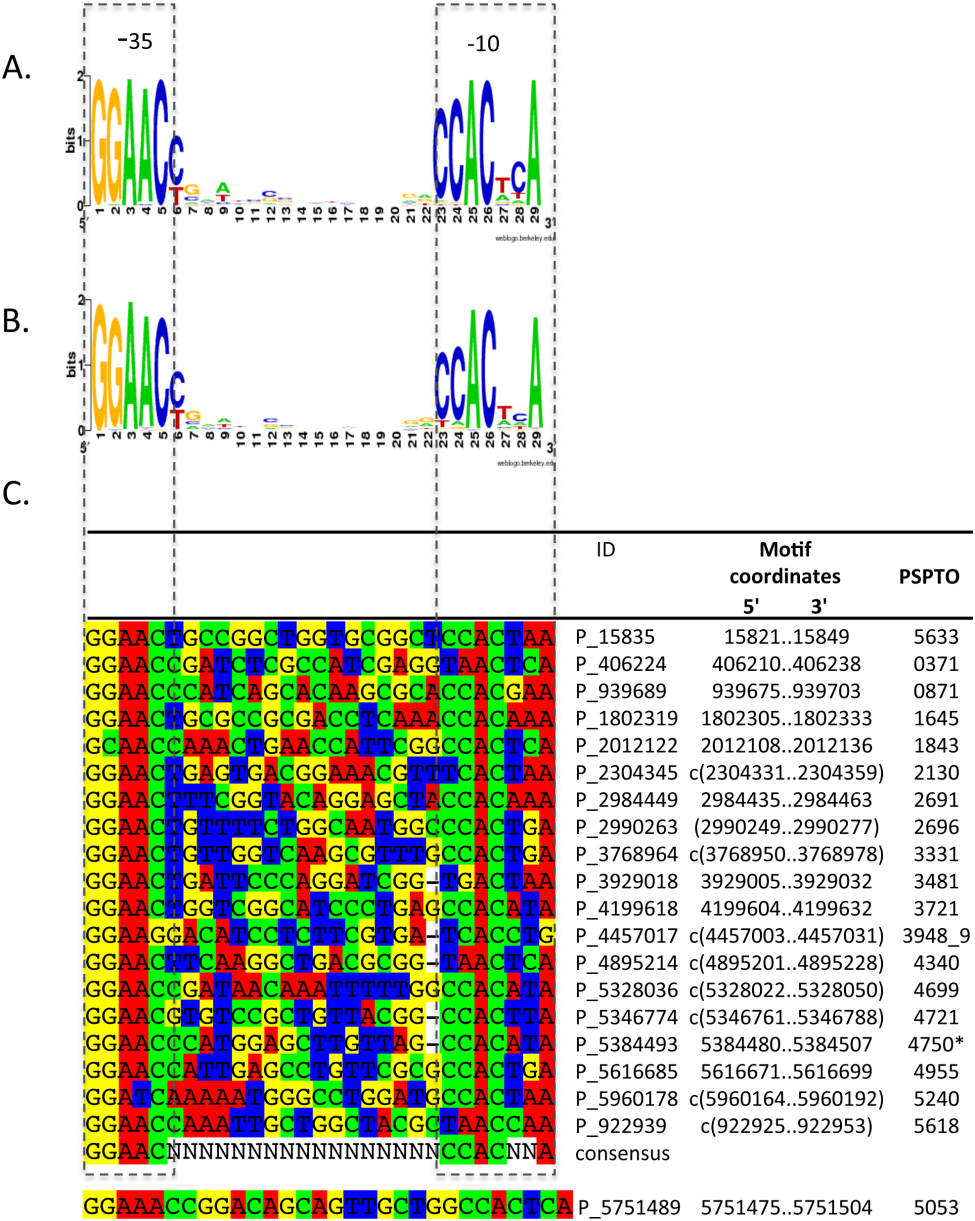
*hrp* promoter sequence alignment. (A). Motif logo for annotated *hrp* promoter sequences. (B). Motif logo for all *hrp* promoters including the newly identified set. The –35 region is highly conserved but two cytosines in the –10 region show variability. (C). Alignment of individual motifs sequences. Motif ID is the central position (genome coordinate) for the associated ChIP-Seq zone of enrichment. Genes downstream of *hrp* promoters are identified by PSPTO numbers. (*): Candidate *hrp* promoter is oriented in an antisense direction relative to PSPTO_4750. PSPTO_3948–9: candidate *hrp* promoter is between PSPTO_3948 and PSPTO_3949, which are oriented covergently. Motif logos were created by Weblogo [75]. Sequences were aligned and visualized using SeaView [76].

**Figure 3:**
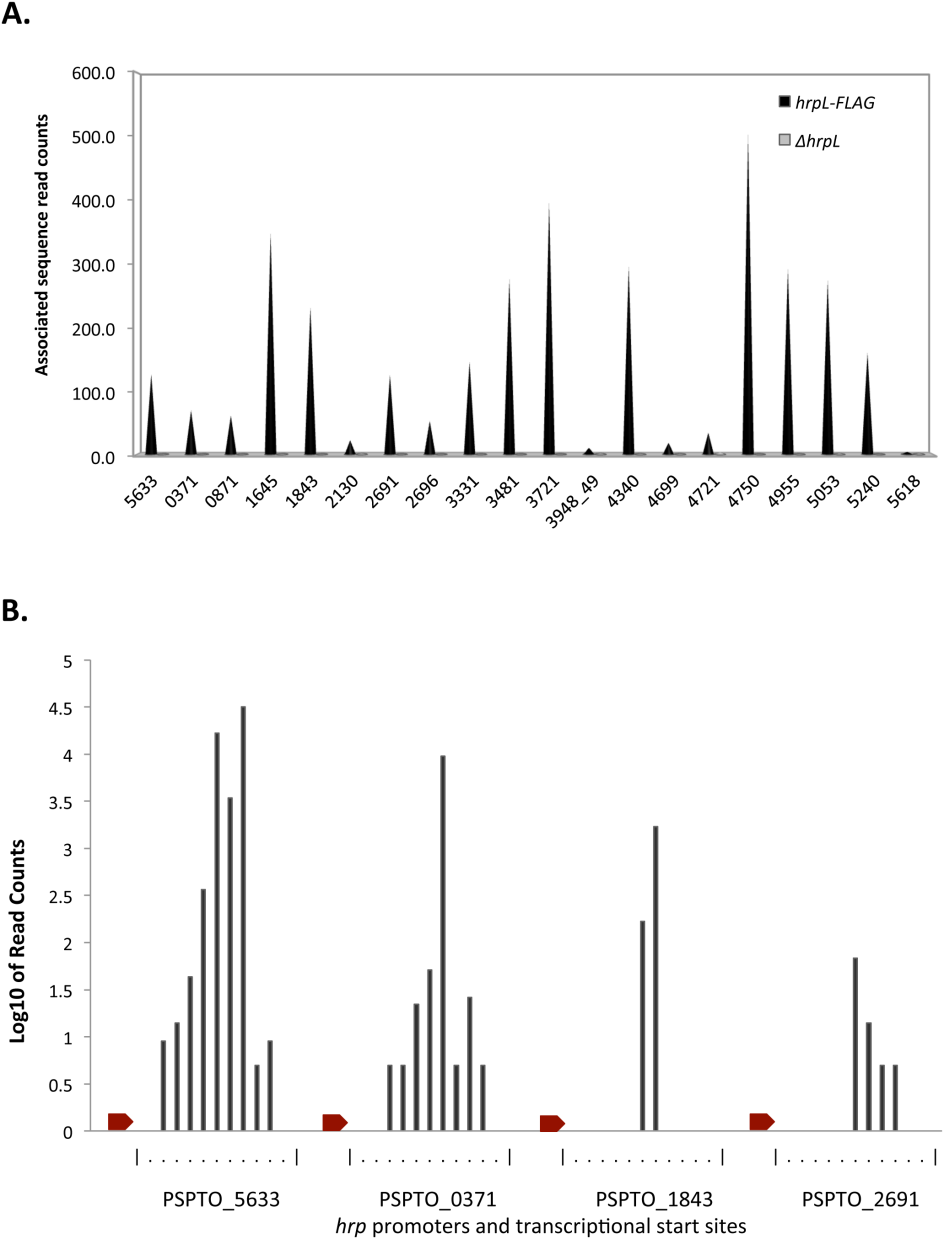
Associated sequence read counts at HrpL-binding sites. (A) The height of each bar corresponds to the number of sequence reads associated with each site enriched by ChIP-Seq, normalized by the number of reads surrounding the peak region (see Materials and Methods). Heights for *hrpL-FLAG* and *ΔhrpL* strains are plotted for comparison. (B) Transcription start site clusters at *hrp* promoters upstream of PSPTO_5633, PSPTO_0371, PSPTO_1843, and PSPTO_2691. Red arrows depict *hrp* promoters. Read counts for captured 5′-ends are shown at positions downstream from each promoter. The mini-scales on the x-axis are positioned so that the leftmost vertical bar corresponds to the 3′ nucleotide of the –10 region in Figure 2 for the corresponding promoter. Dots indicate individual nucleotide coordinates.

Because of the high resolution afforded by ChIP-exo, we were able to align enriched sequences and identify by inspection a conserved motif that resembles the accepted sequence for the HrpL-responsive promoter, GGAAC(–35)-N_16–17_-CCACNNA(–10), particularly in the –35 region (Figure 2). The region upstream of PSPTO_5053 (ID: P_5751489) appears to have an atypical –35 region (GGAAAC) and is longer than the others by 1 nucleotide. This subtle change at –35, however, can be tolerated by extracytoplasmic function (ECF) sigma factors, of which HrpL is an example [55], [56]. Associated sequence read counts at HrpL-binding sites were also computed to represent magnitude of enrichment (Figure 3A). All enrichment values from the *hrpL-FLAG* strain (ranging from 4.7 for P_922939 to 502.0 for P_5384493) are typically much larger than the corresponding values from the *ΔhrpL* strain (0.0 for most cases; 4.0 for P_5346774). The resemblance of the candidate promoters to the canonical *hrp* promoter consensus sequence, together with the evidence that HrpL binds at their genomic locations suggests that they are genuine HrpL-responsive promoters.

### Genome-wide identification of TSSs in RNA-Seq

Active promoters should be associated with nearby transcription start sites (TSSs). Using high-throughput sequencing methods, we mapped TSSs by modifying a stranded RNA-Seq protocol to enrich for primary mRNA transcripts (i.e., those bearing a 5′-triphosphate group) [46]. A unique 18 nucleotide tag was ligated to the 5′ end primary transcripts so that they could be readily identified (see Materials and Methods). The resulting RNA-Seq data contain both whole transcriptome and TSS information (Table 2).

To identify conserved motifs associated with TSS, the 1500 tagged ends with the most abundant reads were selected for analysis. We extracted 50 nucleotides directly upstream from each captured 5′-end, resulting in 1451 sequences derived from the *hrpL-FLAG* sample and 1472 sequences from the *ΔhrpL* sample (overlapping sequences within a sample were merged) and used the sequences as input to MEME [51]. Three motifs with structures resembling canonical promoters were detected in the *hrpL-FLAG* sequences (Figure S1). These include likely RpoD (motif 1, 5′-ttG-N_20_- TANaaT-3′) [33] and RpoF (motif 2, 5′-TaAaG-N_15_-GcCGAta-3′) [57] promoters as well as the putative *hrp* promoter (motif 3, 5′-GgAACc –N_16–17_-CCAN-3′) [23]. We also recovered a weak motif resembling the RpoN promoter (motif 4, 5′-TgG-N_10_-TGC-3′) [58]. Two motifs were recovered from the *ΔhrpL* samples, motif 1 (RpoD) and motif 2 (RpoF). The putative RpoF promoters derived in both cases are upstream of genes likely to be regulated by this sigma factor, such as *fliC* (PSPTO_1949), *fliD* (PSPTO_1951), *flgK* (PSPTO_1944), *cheY-2* (PSPTO_1980) and *cheA-2* (PSPTO_1982) (see supplemental data). As expected, the HrpL motif was identified in the *hrpL-FLAG* but not the *ΔhrpL* samples.

The overall number of detected promoter motifs contrasts with results reported by Filiatrault *et al.* [46], who identified nine distinct motifs upstream from captured 5′-ends (including the *hrp* promoter) using the similar MEME parameters but different DC3000 culture conditions and sample preparation methods. However, in agreement with that report, we also observed that captured ends tend to occur in tightly spaced clusters (see example in Figure 3B). TSS clustering has been noted in other bacteria [59], [60] and in DC3000 for both PvdS and HrpL-associated promoters [46]. The most abundant TSS signal, as well as the signal closest to each promoter candidate, is shown in Table S5. In bacteria, transcription begins 10 bps or less downstream from −10 promoter elements [61]. In four cases the distance between a *hrp* promoter candidate and its closest captured 5′-end exceeds this limit (candidates upstream of PSPTO_1022, PSPTO_A0005, PSPTO_A0012, and PSPTO_B0078). Although these promoters may simply be inactive, it is also possible that the TSSs are undetectable because they are rapidly degraded in the cell or during sample processing. Previous experimental evidence suggests that all four of these *hrp* promoters are genuine [21]–[23]. Another *hrp* promoter candidate, between PSPTO_3948 and PSPTO_3949, is approximately 30 bps distant from the closest TSS. Validation tests suggest that this promoter supports HrpL-dependent transcription (below). In summary, all 20 candidate *hrp* promoters are associated with regions enriched by ChIP-Seq and a plausible *hrp* promoter motif; most are positioned close to captured 5′-ends (between 0–7 bps). It is therefore likely that these represent *bona fide* HrpL-dependent promoters.

### Multiple methods validate candidate HrpL-dependent promoters

The twenty *hrp* promoters discussed above were analyzed further using ChIP-qPCR, reporter fusions and qRT-PCR to confirm HrpL binding, promoter function and HrpL-dependent transcription.

**Figure 4:**
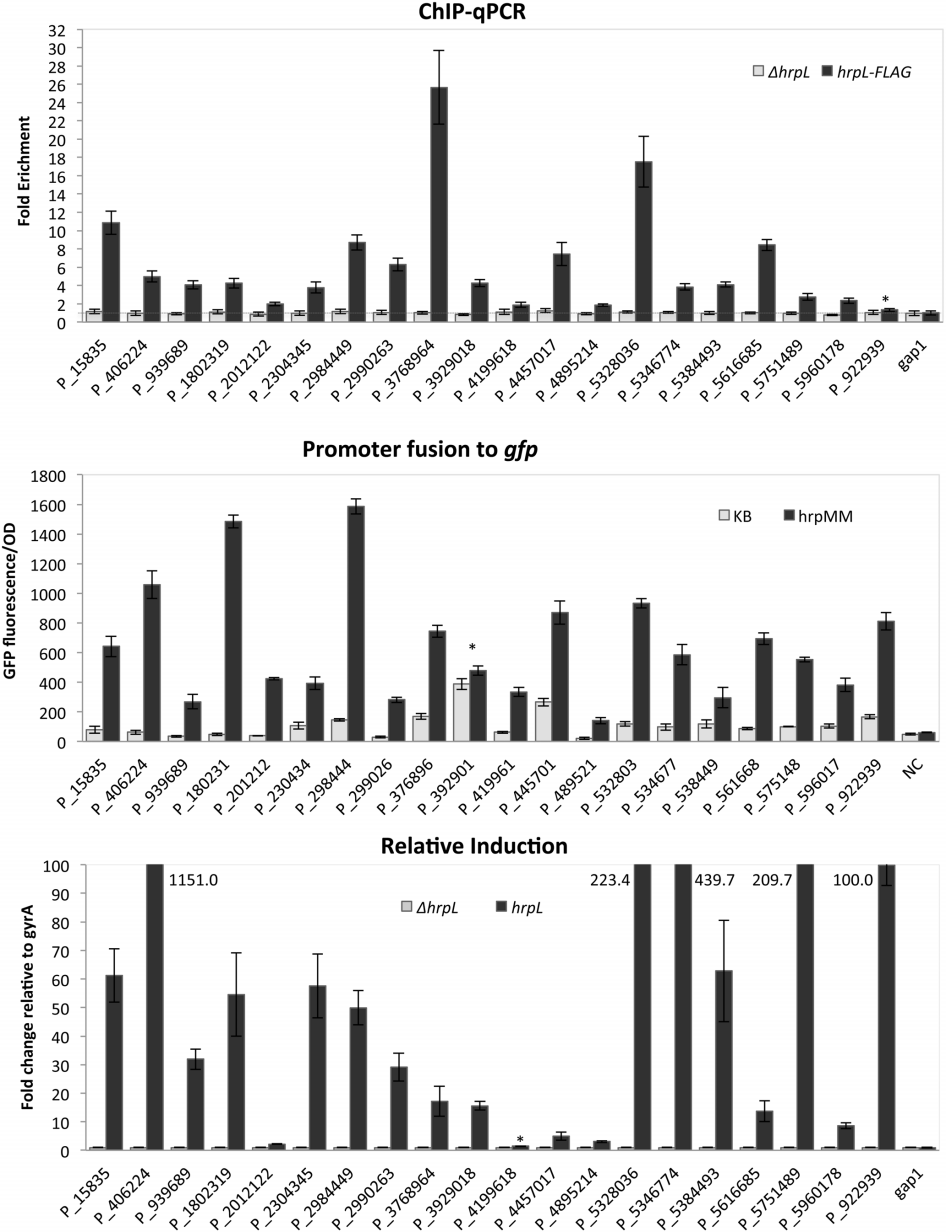
Validation of new *hrp* promoters. (A). ChIP_qPCR experiments to test enrichment of DNA fragments at putative HrpL binding sites. Values for each gene were normalized to results for *gyrA* (DNA gyrase subunit A). *gap-1* (glyceraldehyde 3-phosphate dehydrogenase, type I), not predicted to be HrpL-regulated, was used as a negative control. All fold changes above the expression value for *gyrA* are classified as enriched (above the horizontal line). (B). Induction of cloned *hrp* promoter-*gfp* fusions. Induction was measured by relative fluorescence normalized by OD_600_ (GFP fluorescence/OD) in *hrp*-inducing and *hrp*-repressing conditions. The *hrp* promoter::*gfp* fusion constructs were expressed in the DC3000 Δ*pvsA* siderophore mutant. The promoter trap vector without a promoter insert was used as a negative control (NC). GFP was measured using a Synergy 2 plate reader (Biotech) with excitation from 475 to 495 nm and emission from 506 to 526 nm. OD was measured at 600 nm using the same plate reader. A kinetics reading procedure was used, and a single data point at 5 hours was plotted for all strains, which is the time at which they show a peak value. (C). qRT-PCR analysis showing HrpL-dependent differential expression of transcripts downstream from *hrp* promoters in WT DC3000 and *ΔhrpL* strains. The relative fold change was measured after 1.5 hours on MG supplemented with iron (50 µM final concentration) normalized to *gyrA.* For determination of the relative expression, expression of each gene in the ΔhrpL mutant was set to 1. Expression of each gene in the WT strain was then normalized to the corresponding gene in the ΔhrpL mutant. All data points are the averages of 3 replicates with standard deviations.

Cells for ChIP-qPCR were cultured and collected independently from those used for ChIP-Seq. Analysis of the DNA isolated by immunoprecipitation confirmed enrichment at 19 out of 20 targets (Figure 4A). The exception was P_922939, located upstream of PSPTO_5618 (pseudogene for HopAT1). This promoter also has a relatively weak signal in the ChIP-Seq experiment. However, PSPTO_5618 was transcribed in a HrpL-dependent manner in our other tests (described below).

A plasmid-based reporter trap assay was designed to test promoter activity at defined chromosomal regions and to isolate promoter activity from potentially confounding effects such as read-through from transcription initiated further upstream. Genomic fragments of 100 to 200 nucleotides containing candidate promoters were used to construct transcriptional fusions with a green fluorescent protein (GFP) reporter. Although the basal expression of each promoter was different on KB, all twenty regions showed strong induction in *hrp*-inducing medium compared to KB (Figure 4B). P_3929018 (PSPTO_3481) exhibits robust expression on both rich medium and *hrp*-inducing medium, suggesting that the cloned region contains a promoter that is constitutively expressed. However, qRT-PCR shows that transcription of PSPTO_3481 is 15-fold higher in DC3000 vs. *ΔhrpL* (below). A *hrp* promoter motif at this location was identified in an earlier global transcriptional map [46]. It is possible that this region contains HrpL-dependent and independent promoters.

A qRT-PCR assay was used to assess HrpL-dependent transcription in regions downstream from candidate *hrp* promoters in their native genomic context. Relative transcript abundance for candidates was computed by comparing relative transcript levels at 1.5 hours after shifting bacterial cells from KB to MG (iron supplemented) medium, normalized to mRNA levels for *gyrA*, a housekeeping gene (Figure 4C). Most previously reported *hrp* promoters were associated with strong induction in this experiment (Figure S2). When the relative induction of transcripts in the *ΔhrpL* mutant is compared to that of WT DC3000 in *hrp-*inducing medium, genes downstream from 18 of the new promoter candidates show a strong HrpL-dependent expression. P_406224 (upstream of PSPTO_0371) showed the largest induction in this experiment (>1000x), with four others showing levels ≥100x. P_2012122 (upstream of PSPTO_1843) shows only small differences in mRNA levels between the two backgrounds. P_4199618, upstream of PSPTO_3721, showed no significant induction using qRT-PCR but did exhibit HrpL-dependent behavior in the reporter trap assay. This promoter may depend on other factors for transcription or may ordinarily function at a very low level of activity.

P_5384493 was verified in all experiments. This *hrp* promoter candidate is in an intragenic region downstream of PSPTO_4750 (a hypothetical protein) but is oriented in the antisense direction. As shown in Figure S3, the captured 5′ TSS in close proximity to this promoter is consistent with antisense transcription. Although our qRT-PCR protocol cannot distinguish between transcripts arising from complementary regions, the candidate promoter cloned into our GFP fusion construct showed 2.5 fold induction in *hrp*-inducing compared to KB rich medium.

### Results compared with a computational inventory of *hrp* promoters

Sequence pattern matching has been used extensively to inventory the HrpL regulon in DC3000 [23], [25], [62]. To help determine whether the procedures described above identified all DC3000 *hrp* promoters, we scanned the DC3000 genome using a hidden Markov model trained using previously annotated and new *hrp* promoter sequences (Table S2) and compared the results to those obtained by ChIP-Seq and RNA-Seq. Although the scan matched all annotated and new candidate *hrp* promoters identified in this study, the model did not match any other region in the genome that showed enrichment in the ChIP-Seq experiment (E-value cut-off = 0.001, 245 promoter candidates in total). As a further precaution, the E-value threshold for a match was reset to 0.01, an even more relaxed level that predicts 424 *hrp* promoters in the DC3000 genome. Among matches that are unassociated with confirmed promoters, five are in regions where ChIP-Seq shows weak enrichment (Table S4). These genes are not associated with detectable TSSs within an appropriate distance. The *hrp* promoter-like sequence upstream of PSPTO_0816 (Type IV pilus biogenesis protein) was examined more closely. This candidate shows no significant enrichment using ChIP-qPCR, and does not demonstrate HrpL-dependent transcription using qRT-PCR (data not shown). However, a transcriptional promoter fusion involving this region exhibits a 10.9 fold induction in *hrp*-inducing medium (Table S4). The candidate *hrp* promoter is similar to the canonical *hrp* promoter within the –35 region, but varies at two bases within the –10 region (CAACCAA instead of CCACNNA; Table S4). While it is possible that the induction is due to this promoter candidate rather than some other cryptic promoter, the data are equivocal and the candidate has not been included in Table 3. We suspect that other candidates identified using extremely relaxed criteria will be similarly difficult to classify with confidence.

### The new members of the DC3000 HrpL regulon are largely unrelated to virulence

The annotated functions of the genes associated with the new *hrp* promoters do not appear to involve the T3SS machinery or add to the effector repertoire, with the possible exception of PSPTO_5633 (see below). In order to determine whether the new genes contribute to pathogenicity, we constructed deletion mutants (in WT DC3000 and *ΔhopQ1-1* backgrounds) for seven candidates whose annotated functions were suggestive of plant association and examined them for an altered virulence phenotype in *N. benthamiana* (PSPTO_5633, PSPTO_0371, PSPTO_2691, PSPTO_2696, PSPTO_3331, PSPTO_5240 and PSPTO_2130). No phenotypic differences were observed for *in planta* growth, virulence, or HR for any strain (data not shown). The result for PSPTO_2130 is consistent with another analysis reported for this gene [26]. Although it is not uncommon for effector mutants to fail to exhibit a phenotype (due to functional redundancy [28], [29], [63]), most of these genes are unlikely to be effectors (see Discussion). It is possible that some of the remaining 13 candidates will demonstrate phenotypes if mutated and tested.

### PSPTO_5633 appears to be a weak Type III secretion effector

**Figure 5:**
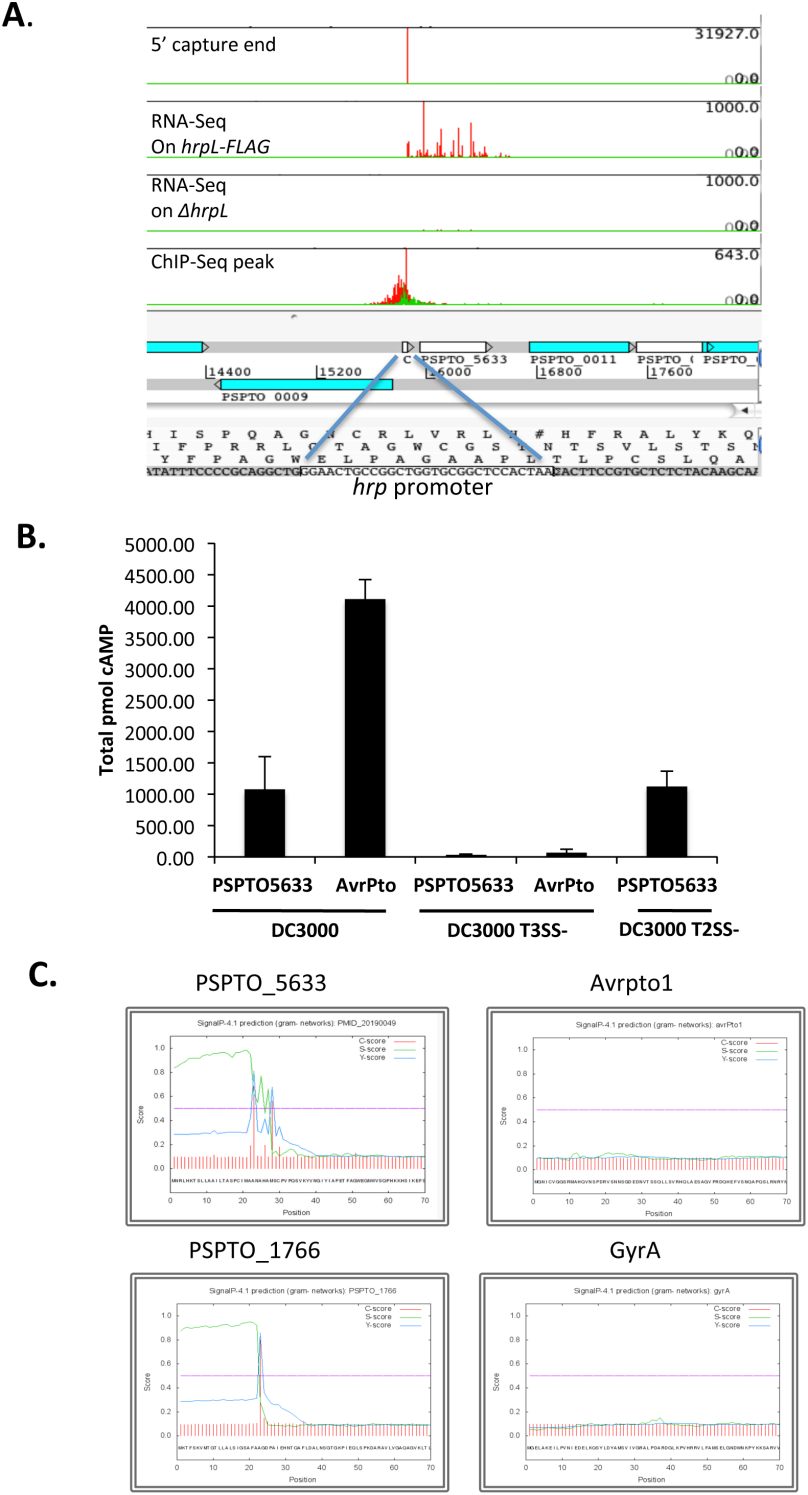
Summary of data for PSPTO_5633. (A). ChIP-Seq, RNA-Seq and promoter motif at PSPTO_5633 locus. The transcription start site mapped by 5′ capture in RNA-Seq and its location relative to the predicted motif are consistent with the presence of a genuine *hrp* promoter. The profiles, along with genome annotation, are shown using Artemis. Red and green traces correspond to sequence read counts on the positive and negative strands, respectively. The sequence containing the *hrp* promoter motif is enclosed in a box. (B) Evidence that PSPTO_5633 is translocated through the DC3000 T3SS. *N. benthamiana* leaves were infiltrated with 5×10^7^ CFU/ml of the indicated DC3000 strains carrying plasmids in which PSPTO_5633 was fused to the Cya translocation reporter, or an AvrPto-Cya control. Total cAMP produced as a result of Cya activity in leaf extracts 6 hours after infiltration is shown for all the strains. PSPTO_5633 is translocated into leaf cells from wild-type DC3000 (T3SS^+^) and from a DC3000Δ*gspD* (T2SS^−^ mutant. No translocation was observed in the DC3000Δ*hrcQ-U* (T3SS^−^ mutant) background. The data represent the average cAMP (pmol) with standard deviations computed using data from 3 plants. The experiment was repeated 3–5 times for all strains except for PSPTO_5633(DC3000 T2SS^−^), which was repeated twice. (C) SignalP analysis showing C, S and Y scores for each position in the sequence of PSPTO_5633, where C-score is the raw cleavage site score, S-score is the signal peptide score and Y-score is the combined cleavage site score. Similar analyses for avrPto1 (a T3SS-translocated effector), PSPTO_1766 (lipase, generally known to target the Sec pathway), and a housekeeping gene (gyrase, generally known to function inside bacterial cells) are shown for comparison.

Although PSPTO_5633 has no annotated function, it shares high sequence similarity with hypothetical proteins in some pathogenic bacterial species (such as *P. syringae* pv. *maculicola*, *Erwinia tracheiphila*, *Citrobacter rodentium, Burkholderia phymatum, Xanthomonas campestris* and *Yersinia mollaretii*), which is frequently the case for an effector. However, the TEREE (Type III Effector Relative Entropy Evaluation) score for this gene is –2 [64], which is outside of the range exhibited by most effectors (–13 to –47). Nonetheless we investigated PSPTO_5633 as a potential effector. Figure 5A shows PSPTO_5633 and its neighboring *hrp* promoter motif, aligned with the ChIP-Seq, RNA-Seq and 5′-capture profiles in this genomic region.

As effectors need to be transported into the plant cytoplasm to interact with plant defenses, we tested PSPTO_5633 for its ability to translocate into plant cells. Figure 5B shows that PSPTO_5633 enters plant cells in a T3SS dependent manner. PSPTO_5633 translocation is weak when compared to that for AvrPto but is significantly above background, a result supported by multiple experimental replicates. Based on these results, PSPTO_5633 has been assigned the name HopBM1 to recognize its role as an effector.

A closer look at the N-terminal region of PSPTO_5633 surprisingly suggests that this protein may be secreted through the Sec pathway (see Figure 5C). To test this possibility, translocation of PSPTO_5633 was assayed in a Δ*gspD* background (T2SS–). The level of cAMP observed using a T2SS–strain was indistinguishable from that seen with DC3000 (Figure 5B), suggesting that PSPTO_5633 does not enter plant cells using this pathway.

### Comparative genome analysis of the HrpL regulon

**Figure 6:**
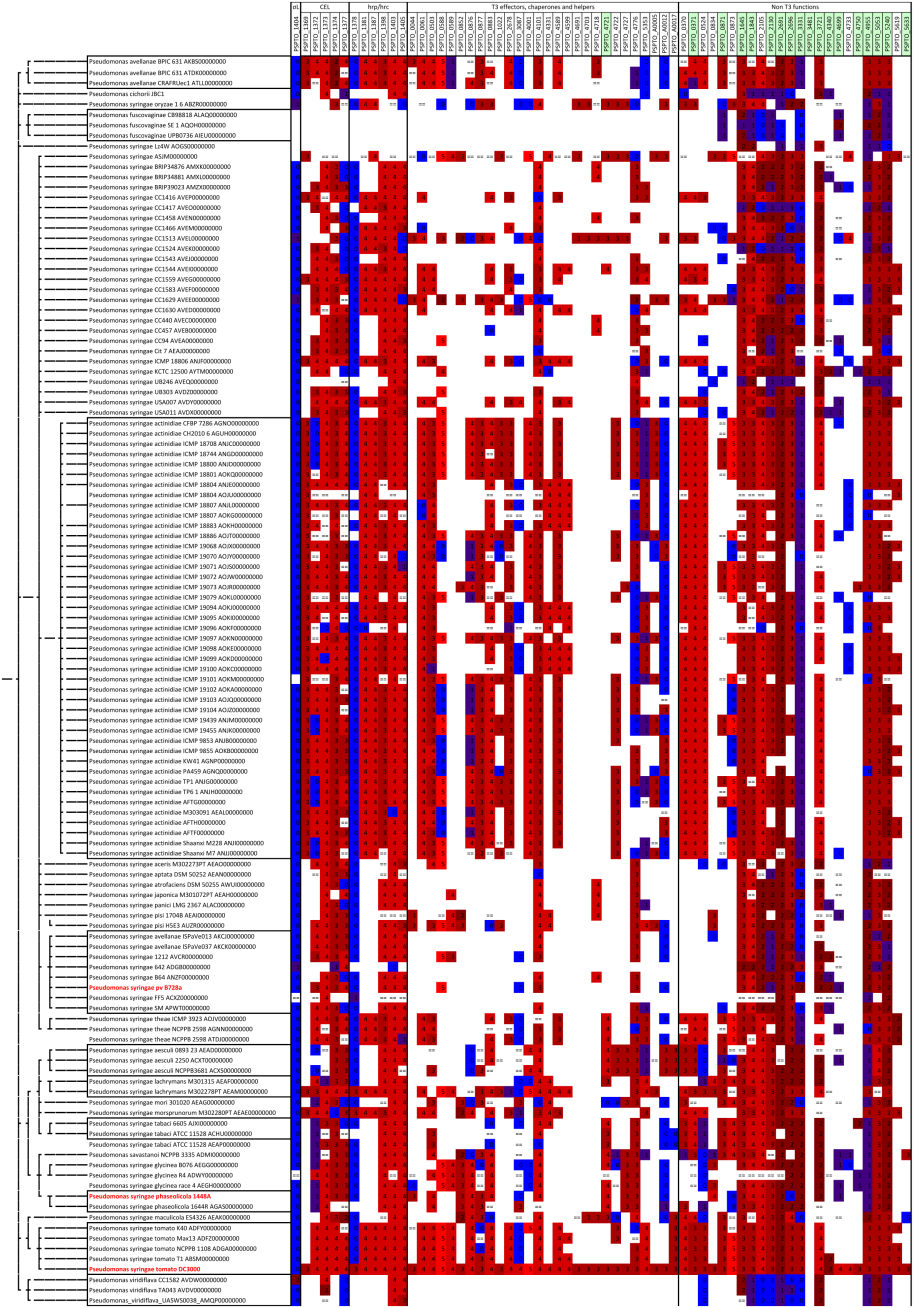
Orthologs and *hrp* promoter motifs for DC3000 HrpL regulon orthologs in the *P. syringae* subgroup. A blank (white) cell indicates that no ortholog was detected. “ =  = ” indicates that an orthologous gene was identified but no upstream sequence could be extracted (due to incomplete sequence information and segmentation in draft genomes). For cases in which orthologs were detected and upstream sequences recovered, the color represents the –logarithm (base 10) of the HMM E-value for the best motif matching the *hrp* promoter model in the upstream sequence. A continuous color scheme is used where blue represents a poor match (E-value = 1), dark red indicates an intermediate match (E-value = 1e-02), and bright red indicates a good match (E-value 1e-05). Most verified *hrp* promoters in DC3000 match with values above 3. The leftmost gene column represents orthologs for the HrpL sigma factor, PSPTO_1404. In DC3000, this sigma factor is transcribed from a RpoN-responsive promoter [77]. Genes immediately downstream of *hrp* promoters are shown in columns, as they appear in *CEL*, *hrp/hrc* cluster, followed by type III effectors, chaperones and helpers, and non-type III function genes. Newly found members are in green background. 3-color scale is used: Color: Blue …… dark red …… light red Value: 0…….…………2………….…….5.

Although a sigma factor regulon can be described as a tightly integrated collection of genes, the composition of the HrpL regulon across species is not rigid. While the core effector delivery system is highly conserved, the effector proteins delivered by it vary considerably [65] in other *P. syringae* pathovars. In order to determine whether the new HrpL regulon members show similar patterns of conservation, we conducted an *in silico* analysis in which we examined genome sequences from 121 members of the *P. syringae* group, most of which are plant pathogens. The group (taxid 136849) is defined by NCBI in their taxonomy database (http://www.ncbi.nlm.nih.gov/taxonomy). We identified orthologs to the DC3000 genes immediately downstream from the 73 *hrp* promoters and then examined the DNA sequences upstream from them for patterns matching the *hrp* promoter (Figure 6). As expected, nearly all of the *P. syringae* group genomes contain HrpL orthologs. *hrp* promoter motifs are observed upstream of most other regulon orthologs (bright red squares) including those corresponding to the new regulon members (shown with names on a green background). Genes for core T3SS functions are widely shared across the group, whereas orthologs for the DC3000 effector genes are not as conserved, reflecting effector diversity. About half of the new regulon members are as conserved as the core T3SS genes. Others (such as PSPTO_5633 and PSPTO_3481) exhibit a different pattern and are found only in DC3000 and a few other *P. syringae* genomes (Figure S4). This sparse distribution resembles that found for effectors such as PSPTO_4691 (HopAD1) and PSPTO_4703 (HopAQ1). Note that ortholog absence should be interpreted cautiously since it can reflect errors in genome assembly or shortcomings in the methods used to identify reciprocal best BLASTP matches, as well as the actual absence of an ortholog in a genome.

**Figure 7:**
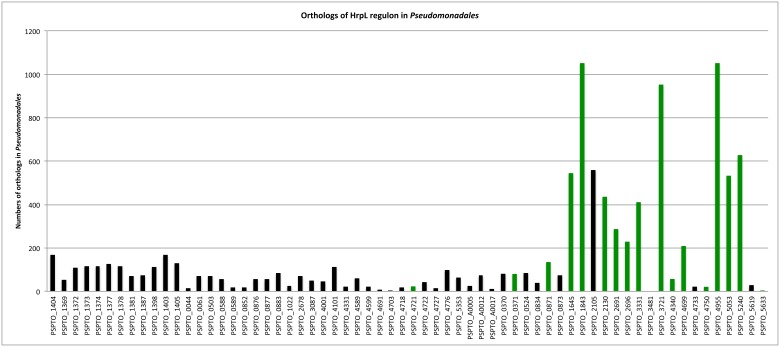
Ortholog inventory of HrpL regulon in *Pseudomonadales*. Green represents newly found members; black represents previously annotated regulon members. The values shown represent counts of orthologs of HrpL regulon members across 1060 species.

A substantially different result is obtained when the analysis is extended to all *Pseudomonadales* genome sequences (1060 organisms total), most of which are neither plant pathogens nor contain HrpL orthologs (Table S3). Orthologs for core regulon components are rarely detected outside the *P. syringae* group. In contrast, several new regulon members are widely represented (Figures 7 and S4). Examples are PSPTO_1843 (aspartate kinase), PSPTO_3721 (enoyl-[acyl-carrier-protein] reductase) and PSPTO_4955 (thiosulfate sulfurtransferase/phosphatidylserine decarboxylase) whose orthologs appear in almost all sequenced strains tested.

## Discussion

Genome-wide approaches are well suited for investigating sigma factor regulons containing multiple chromosomally dispersed genes and operons [42], [45], [66], [67]. Several attempts have been made to define the set of genes regulated by HrpL in DC3000 [21]–[23] and these have succeeded in identifying many effectors and essential components of the T3SS. Although these efforts were intended to be thorough [24], [25], the availability of more advanced methodologies makes it both feasible and worthwhile to conduct a new inventory. The power of this approach was recently demonstrated in a report describing the use of RNA-Seq to link the expression of HrpL to members of the HrpL regulon in six different *P. syringae* isolates [26].

Using a combination of high-throughput sequencing and computational analyses, we have searched intensively for members of the HrpL regulon in DC3000 by identifying and confirming likely *hrp* promoters. The results increase the number of HrpL-responsive promoters in DC3000 to 73, including 52 of the 54 promoters already annotated in the DC3000 genome. The experiments reported here establish two important molecular details relevant to regulation by HrpL. First, candidate HrpL binding sites were defined by their immuno-co-precipitation with a FLAG-tagged version of HrpL, using both ChIP-Seq and more localized immune-precipitation experiments to confirm the ChIP-Seq results. All of these regions contain a conserved motif that closely resembles the known *hrp* promoter sequence. Evidence for HrpL binding at these sites has not been reported previously. Second, we show that many of these motifs are active promoters. They are closely associated with transcription start sites, defined using RNA-Seq to capture mRNA 5′-ends that map to genomic coordinates immediately downstream of the putative promoters. Using plasmid-based reporter systems, we demonstrate that cloned regions containing most of the promoter candidates also support transcription in a HrpL-dependent manner, and we can also detect HrpL-dependent transcription from regions downstream from the promoters in their native locations using qRT-PCR. Together, these experiments strongly support the addition of the new *hrp* promoters to the currently annotated set as well as the addition of the genes downstream of them to the HrpL regulon.

### Integration of ChIP-Seq and RNA-Seq data with earlier DC3000 HrpL regulon inventories

Many previously reported *hrp* promoters have been experimentally validated [21]–[23], [26]. Transcription start sites have also been globally mapped onto the DC3000 genome, including those associated with likely HrpL-dependent promoters [46]. Combining our data with these, we can summarize our current understanding of *hrp* promoters in DC3000 along with relevant evidence (Table 3 and Table S5).

All previously reported *hrp* promoters were confirmed with three exceptions. We saw no evidence for HrpL-binding upstream of PSPTO_3489 (sugar ABC transporter/ATP-binding protein), classified by Ferreira *et al.* [23] as a HrpL regulon member. A *hrp* promoter was also predicted within PSPTO_1369 (*shcN*) upstream of PSPTO_1370 (virulence factor HopN1) [21]–[23]. A TSS was detected three nucleotides downstream from this promoter (Figure S3), but other evidence suggests that PSPTO_1369 and PSPTO_1370 are co-transcribed from a *hrp* promoter upstream of PSPTO_1369, where HrpL binding is very clearly observed (Figure S3). Finally, although a *hrp* promoter was reported upstream of *PSPTO_3576* (*tvrR*, TetR-like virulence regulator) in an earlier 5′-capture experiment [46], we did not observe enrichment of the region containing it in our ChIP-Seq screen. The activity of this promoter was also previously shown to be independent of HrpL in a promoter trap experiment [68].

We found evidence for three *hrp* promoters that had been noted previously but described as weakly supported. A putative promoter upstream of PSPTO_1645, reported by Ferreira *et al.* [23], was not included in their high-confidence list because the distance from the translational start site of the gene to the predicted promoter was too large. In contrast, we found evidence for HrpL-binding at this site and a TSS in close vicinity (3 to 7 bps from the 3′-end of the −10 region) (Table S5). The same authors report that a second gene, PSPTO_2691, was induced by HrpL but had no obvious *hrp* promoter associated with it. However, we detected HrpL binding, a captured TSS, and a *hrp* promoter motif upstream from this gene. PSPTO_2691 was also recently identified as a HrpL regulon member by Mucyn *et al.* [26]. Finally, the *hrp* promoter upstream of the *hopAT1* pseudogene (PSPTO_5618) was noted in [23] but was not included in the DC3000 annotation. Although the binding activity of HrpL at this location was not strong (Figure 4A), this region generated a positive result in the promoter fusion assay (Figure 4B) and transcription of the pseudogene was also elevated in the presence of HrpL in the qRT-PCR assay (Figure 4C).

A comparison between the results presented here and those reported recently by Mucyn *et al.* [26] reveals many similarities. Both studies identified the majority of known HrpL regulon members. In addition, we confirmed six previously classified HrpL regulated genes that were not detected by their survey (PSPTO_0044, PSPTO_1022, PSPTO_4101, PSPTO_4724, PSPTO_5353 and PSPTO_5616; Table S5). With respect to new regulon members, five are shared (PSPTO_0371, PSPTO_0871, PSPTO_2130, PSPTO_2691 and PSPTO_4721). However, the two studies differ significantly when other new regulon candidates are examined. Fifteen new regulon members reported here (including PSPTO_5633; see below), and 12 in Mucyn *et al.*, are not in common. A likely explanation for the disagreement is that Mucyn *et al*. optimized their experiments to identify genes that respond to HrpL either directly or indirectly, and employed a cloned *hrpL* gene under the control of an arabinose-inducible promoter on a multi-copy plasmid. Some genes identified in this fashion would be expected to have no associated HrpL-binding activity or *hrp* promoter motifs in their upstream regions. This appears to be true for the 12 candidates reported by Mucyn *et al.* that are not in agreement (see note in Table 3). In contrast, the experiments reported here were optimized to identify genes directly regulated by HrpL. The *hrpL* gene was expressed from its native location, and candidates were identified by a combination of sigma factor binding, associated *hrpL* promoter motifs, TSS and the demonstration of HrpL-dependent transcriptional activity.

### AprI (PSPTO_3331), an inhibitor of metalloprotease AprA, is regulated by HrpL

In *P. aeruginosa*, the protease AprA (orthologous to PSPTO_3332) is secreted by the T1SS and degrades flagellin monomers [69]. Recently, Pel *et al*. [70] analyzed this factor and its role in DC3000. Their results demonstrate that AprA is required for full virulence in DC3000 and functions by degrading flagellin before it can trigger plant defense mechanisms via the plant receptor FLS2. Although their work establishes that AprI can inhibit the protease either in vitro or when it is expressed in transgenic plants, they note that AprI is probably delivered to the bacterial periplasmic space by its T2SS signal peptide [71]. It is therefore unlikely to encounter the protease, making its function unclear.

Our results draw additional attention to this important system by establishing that *aprI* is downstream from a functional *hrp* promoter and is therefore a member of the HrpL regulon. The identity of the promoter is strongly supported by several lines of evidence (Table 3). An earlier transcriptome analysis identified a captured 5′-end and a potential RpoN promoter motif at this position [46] but we are unable to detect RpoN binding to this region in a ChIP experiment (data not shown). Filiatrault *et al*. also observed a captured 5′-end upstream from *aprA*, possibly linked to an RpoD promoter. The two genes, which are 59 bps apart, therefore appear to be regulated independently. Why *aprI* is regulated by HrpL is not obvious, especially if it is expressed at the same time as *aprA* (i.e., during an infection). It is possible that the target for AprI in the periplasm is another protease unrelated to AprA. However, deletions in *aprI* in either DC3000 or ΔQ1-1 backgrounds exhibit no growth or virulence phenotypes *in planta*, leaving the role of AprI a mystery. AprI orthologs are widespread in the *P. syringe* group (Figure 6) although in some cases without accompanying *hrp* promoter motifs. It is also found in other pseudomonads including *P. fluorescens* (Table S5).

### PSPTO_5633 (HopBM1) is a weak effector

Three characteristics common to most known *P. syringae* effectors are transcription via HrpL, the appearance of effector homologs in other pathogens, and an N-terminal protein sequence with certain characteristic features [64]. The N-terminal features include multiple serine residues within the first 50 amino acids, an aliphatic residue (isoleucine, valine, leucine, alanine, methionine) or proline at positions 3 or 4, and a lack of acidic amino acids (aspartic acid, glutamic acid) within the first 12 residues [64]. While new regulon members such as PSPTO_3331 (also discussed above), PSPTO_4340 (insecticidal toxin protein) and PSPTO_4699 (a non-ribosomal peptide synthetase component) satisfy the N-terminal criteria for T3SS substrates, their annotated functions suggest that they are not effectors.

PSPTO_5633 did not appear in the original DC3000 annotation but was added following a global transcriptome analysis [30]. PSPTO_5633 shares high sequence similarity with hypothetical proteins in three members of the *P. syringae* group (Figure 6) as well as with proteins in several other pathogenic bacterial species (mentioned above). However, its leader sequence scores poorly when it is examined for the amino acid patterns mentioned above [64]. In addition, DC3000 plasmid gene PSPTO_B003, identical in sequence to PSPTO_5633, was reported to have no T3SS translocation activity in a *P. fluorescens* background [72]. Our data demonstrate that PSPTO_5633 is detectably but weakly translocated into plant cells in a manner that depends on the T3SS (Figure 5B). The disagreement between these results is probably due to the fact that translocation in general is lower in the *P. fluorescens* system than in DC3000 [72]. Since PSPTO_5633 translocates poorly in DC3000, its translocation may be undetectable in *P. fluorescens*.

An analysis of the N-terminal region of PSPTO_5633 using SignalP [73] suggests that it may be secreted through the Sec pathway (Figure 5C). Other proteins within the HrpL regulon share this characteristic, including PSPTO_3331 (newly found), PSPTO_0524, and hopAJ1. Our results are not consistent with a model in which PSPTO_5633 is delivered through the T2SS, either directly or indirectly, into plant cells (Figure 5B). However, since this experiment depends on the interaction of Cya with calmodulin within the plant cell, it does not test secretion through the T2SS *per se*. Additional experiments will be required to determine whether PSPTO_5633 or other HrpL regulon members are T2SS substrates.

### HrpL regulon members are represented in other pseudomonadales

The new HrpL regulon members are largely conserved within the *P. syringae* group, albeit at varying levels (Figure 6). Table S3 summarizes the orthology analysis for all 1060 *Pseudomonadales* genomes. Although the large size of this data set precludes a detailed analysis, broad patterns can be discerned. Orthologs for the core regulon components such as *hrpL*, *CEL*, *hrp/hrc* and effector genes are infrequently found outside the *P*. *syringae* group. However, several new regulon members are widely represented (Figure 7). Examples are PSPTO_1843 (aspartate kinase), PSPTO_3721 (enoyl-[acyl-carrier-protein] reductase) and PSPTO_4955 (thiosulfate sulfurtransferase/phosphatidylserine decarboxylase) whose orthologs appear in almost all sequenced strains tested. Interestingly, in some cases these are accompanied by upstream *hrp* promoter motifs even in genomic contexts where HrpL is absent, although this is relatively uncommon.

Diversity in the number of HrpL regulon orthologs and their distinct patterns of conservation across the *Pseudomonadales* imply that HrpL regulon member recruitment is a complex process. A gene is likely to be selected for HrpL regulon membership if its expression confers an advantage in the nutrient-poor and stressful environment of the plant apoplasm, especially when coupled to the expression of the regulon as a whole. The assembly and refinement of a working HrpL regulon probably arises due to multiple evolutionary events, including horizontal gene transfer [5], changes in coding regions that alter or eliminate protein function (e.g. conversion into pseudogenes), and variations in *cis*- acting elements [26] or the factors that recognize them. Promoter alteration may be the easiest recruitment mechanism for inclusion into a regulon [74].

In summary, our analysis revealed 20 new HrpL regulon members. The combination of laboratory and computational methods used here makes it likely that the inventory at this point is nearly complete. One conceptual difficulty in “closing” the list is that relaxed criteria can result in candidates that satisfy one of several tests for membership (such as the apparent HrpL-dependent transcription from a region directly upstream from PSPTO_0816 in a promoter trap assay). We suggest however that evidence for HrpL binding, the presence of a motif, and demonstrated transcriptional activity together best define the regulon in practical terms. The remaining challenge is to determine what roles, if any, the new regulon members play in the process of pathogenesis.

## Supporting Information

Figure S1 **Likely promoter motifs recovered by MEME using 5′-end capture data from** ***hrpL-FLAG*** **and** ***ΔhrpL*** **cells.**
(TIF)

Figure S2 **qRT-PCR analysis showing HrpL-dependent transcription downstream from 38 known HrpL regulon members.** Relative transcript change was compared between DC3000 and *ΔhrpL* strains. Relative induction of each gene was normalized to the housekeeping gene *gap1.* No transcription induction was observed in KB, while significant induction was seen after medium shift to MG supplemented with iron (50 µM final concentration) after 1.5 hr. Values are averages of three replicates with standard deviations.
(TIF)

Figure S3 **ChIP-Seq and RNA-Seq data for selected HrpL regulon members.** The red line represents mapped reads corresponding to the positive strand and the green line shows reads mapped to the negative strand. Genome annotation is shown below profiles. The sequence containing the *hrp* promoter motif is represented by green boxes. The 5′ capture profile from Filliatrault *et al.* [46] is included in some panels for comparison.
(TIF)

Figure S4 **Ortholog inventory of HrpL regulon in** ***P*** **.** ***syringae*** **group.** Green represents newly found members; black represents previously annotated regulon members. The values shown represent counts of orthologs of HrpL regulon members across 121 species.
(TIF)

Table S1 **All primers used in this study.**
(XLSX)

Table S2 ***hrp*** **promoter motif sequences included in the HMM training set.**
(DOCX)

Table S3 **Orthologs and** ***hrp*** **promoter motif scores for HrpL regulon members in 1060** ***Pseudomonadales*** **genomes.** A blank (white) cell indicates that no ortholog was detected. “ =  = ” indicates that an orthologous gene was identified but no upstream sequence could be extracted (due to incomplete sequence information and segmentation in draft genomes). For cases in which orthologs were detected and upstream sequences recovered, the color represents the –logarithm (base 10) of the HMM E-value for the best motif matching the *hrp* promoter model in the upstream sequence. A continuous color scheme is used where blue represents a poor match (E-value = 1), dark red indicates an intermediate match (E-value = 1e-02), and bright red indicates a good match (E-value 1e-05). Most verified HrpL promoters in DC3000 match with values above 3. The leftmost gene column represents orthologs for the HrpL sigma factor, PSPTO_1404. In DC3000, this sigma factor is transcribed from a RpoN-responsive promoter [77]. Genes immediately downstream of *hrp* promoters are shown in columns, as they appear in *CEL*, *hrp/hrc* cluster, followed by type III effectors, chaperones and helpers, and non-type III function genes. Newly found members are in green background. 3-color scale is used: Color: Blue …… dark red …… light red Value: 0…….…………2………….…….5.
(XLSX)

Table S4 **Additional** ***hrp*** **promoter-like sequences with limited experimental support.**
(DOCX)

Table S5 **Data for all** ***hrp*** **promoters.**
(DOCX)

Dataset S1 **S1.1 to S1.6. Artemis-loadable profiles for the DC3000 chromosome, plasmid A and Plasmid B.** These can be used to visualize results for ChIP-exo of *hrpL-FLAG*, t = 1.5 hours (dataset S1.1 to S1.3); ChIP-Seq of *ΔhrpL*, t = 1.5 hours (dataset S1.4 to S1.6), respectively.
(ZIP)

Dataset S2 **S2.1 to S2.6. Artemis-loadable profiles for the DC3000 chromosome, plasmid A and Plasmid B.** These can be used to visualize results for RNA-Seq of *hrpL-FLAG*, t = 1.5 hours (dataset S2.1 to S2.3); ChIP-Seq of *ΔhrpL*, t = 1.5 hours (dataset S2.4 to S2.6), respectively.
(ZIP)

Dataset S3 **S3.1 to S3.6. Artemis-loadable profiles for the DC3000 chromosome, plasmid A and Plasmid B.** These can be used to visualize results for 5′ capture (TSSs) of *hrpL-FLAG*, t = 1.5 hours (dataset S3.1 to S3.3); ChIP-Seq of *ΔhrpL*, t = 1.5 hours (dataset S3.4 to S3.6), respectively.
(ZIP)

Dataset S4 **S4.1, S4.2. gff files containing positions of 1500 top peaks in 5′ capture data of** ***hrpL-FLAG*** **and** ***ΔhrpL*** **samples.**
(ZIP)

Dataset S5 **gff file containing new** ***hrp*** **promoters in DC3000.**
(ZIP)

Dataset S6 **gff file containing results obtained from the HMM scan (p = 0.001) for** ***hrp*** **promoter motifs in the DC3000 genome.**
(ZIP)

Dataset S7 **MEME analysis of sequences upstream from captured 5′-ends using RNA-Seq data from** ***hrpL-FLAG*** **cells at t = 1.5 hours.**
(ZIP)

Dataset S8 **MEME analysis of sequences upstream from captured 5′-ends using RNA-Seq data from** ***ΔhrpL*** **cells at t = 1.5 hours.**
(ZIP)
