# Supplementary figures and images for: Global Analysis of the HrpL Regulon in the Plant Pathogen Pseudomonas syringae pv. tomato DC3000 Reveals New Regulon Members with Diverse Functions
Source: PLoS One. 2014 Aug 29;9(8):e106115. doi: 10.1371/journal.pone.0106115 (PMC4149516; doi:10.1371/journal.pone.0106115)

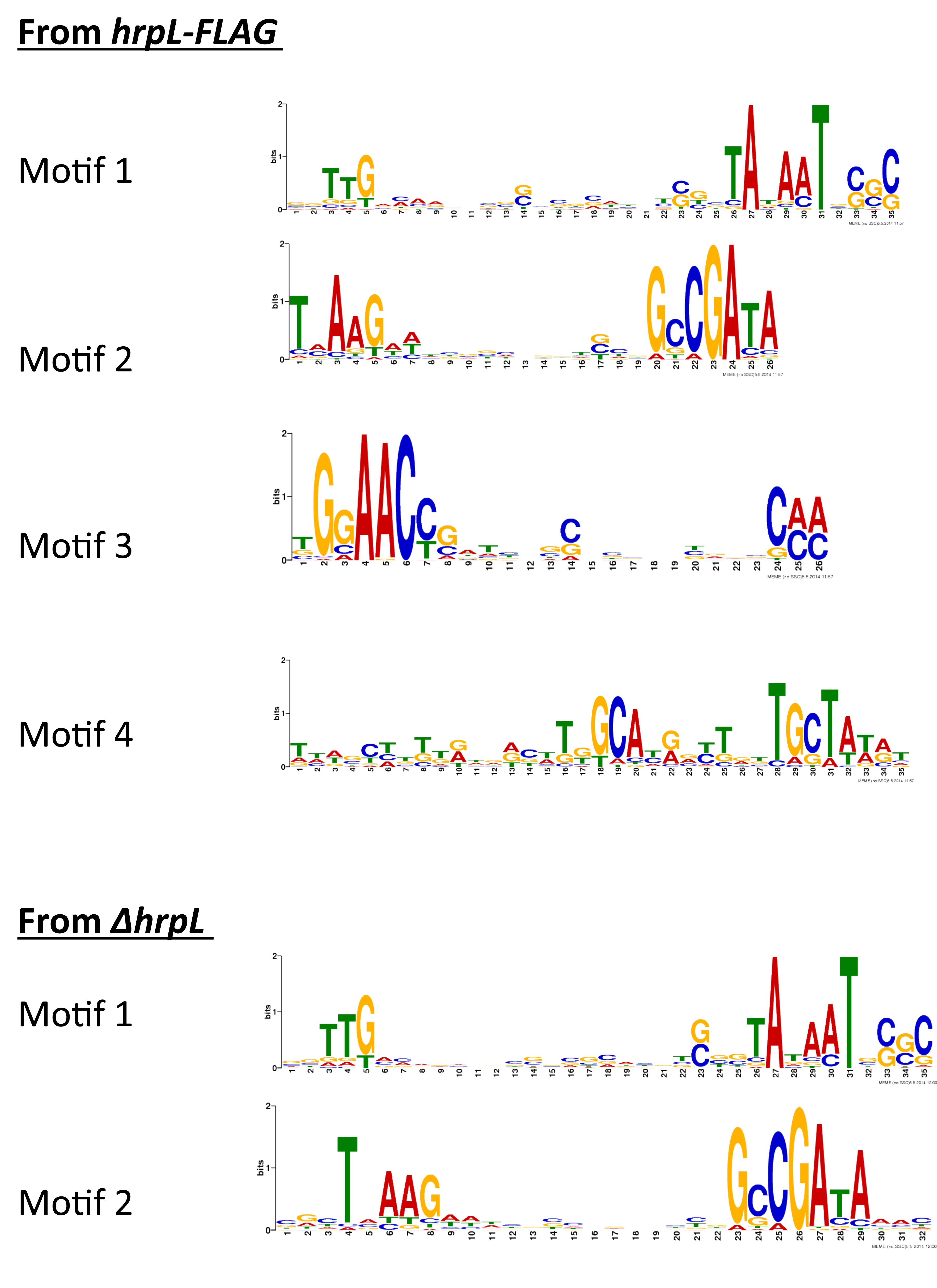

Supplement: Figure S1 — Likely promoter motifs recovered by MEME using 5′-end capture data from hrpL-FLAG and ΔhrpL cells. (TIF) [file pone.0106115.s001.tif]

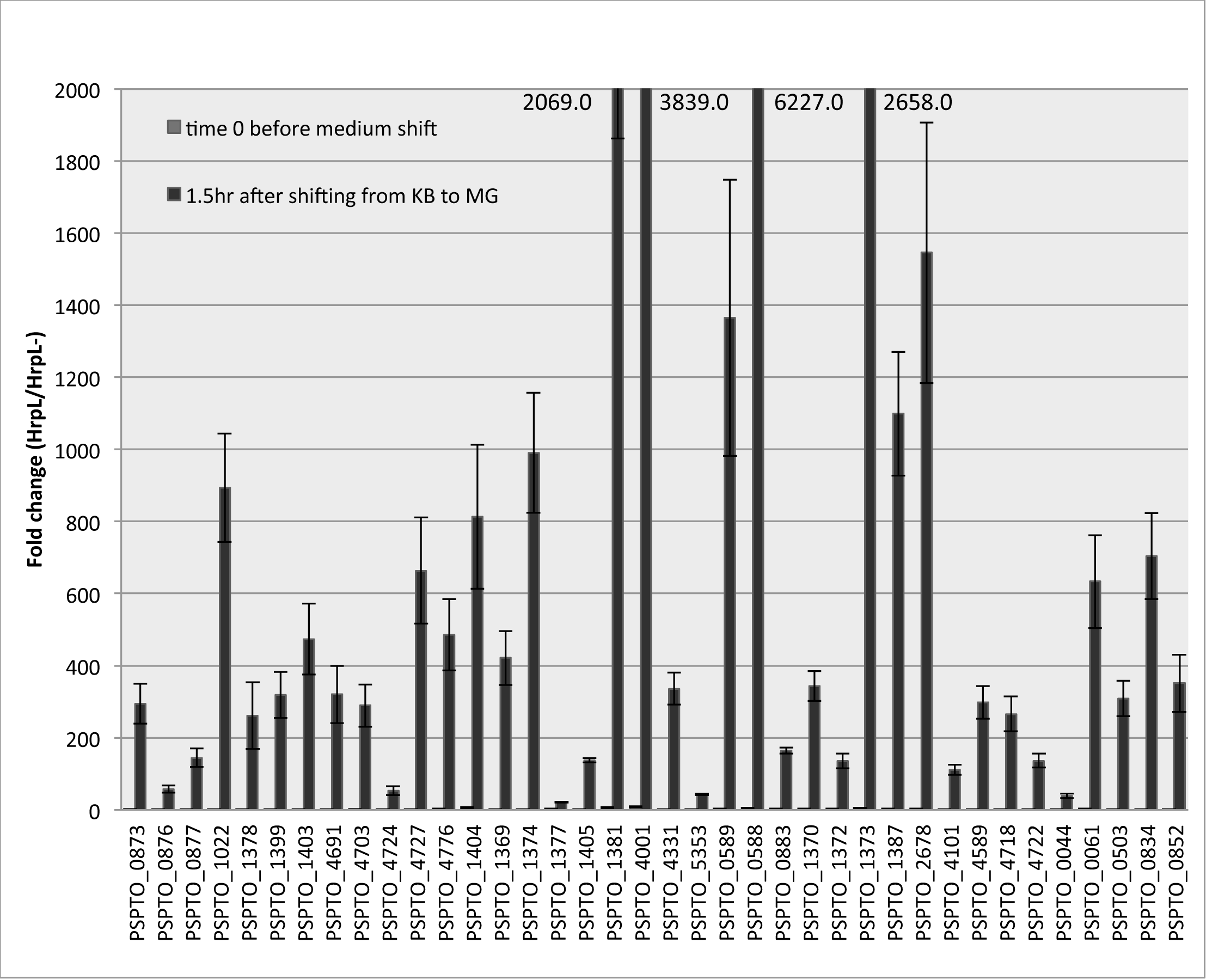

Supplement: Figure S2 — qRT-PCR analysis showing HrpL-dependent transcription downstream from 38 known HrpL regulon members. Relative transcript change was compared between DC3000 and ΔhrpL strains. Relative induction of each gene was normalized to the housekeeping gene gap1. No transcription induction was observed in KB, while significant induction was seen after medium shift to MG supplemented with iron (50 µM final concentration) after 1.5 hr. Values are averages of three replicates with standard deviations. (TIF) [file pone.0106115.s002.tif]

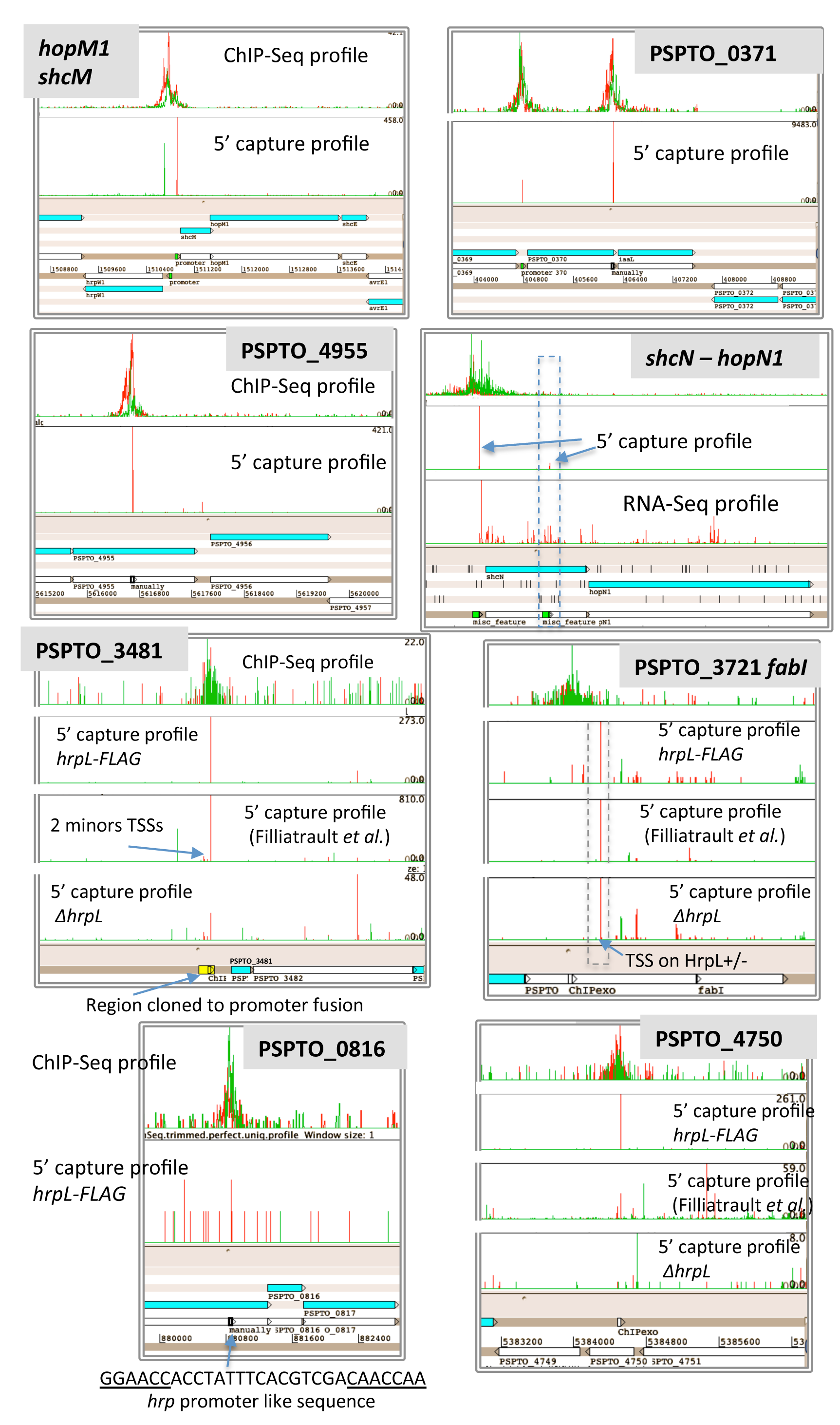

Supplement: Figure S3 — ChIP-Seq and RNA-Seq data for selected HrpL regulon members. The red line represents mapped reads corresponding to the positive strand and the green line shows reads mapped to the negative strand. Genome annotation is shown below profiles. The sequence containing the hrp promoter motif is represented by green boxes. The 5′ capture profile from Filliatrault et al. [46] is included in some panels for comparison. (TIF) [file pone.0106115.s003.tif]

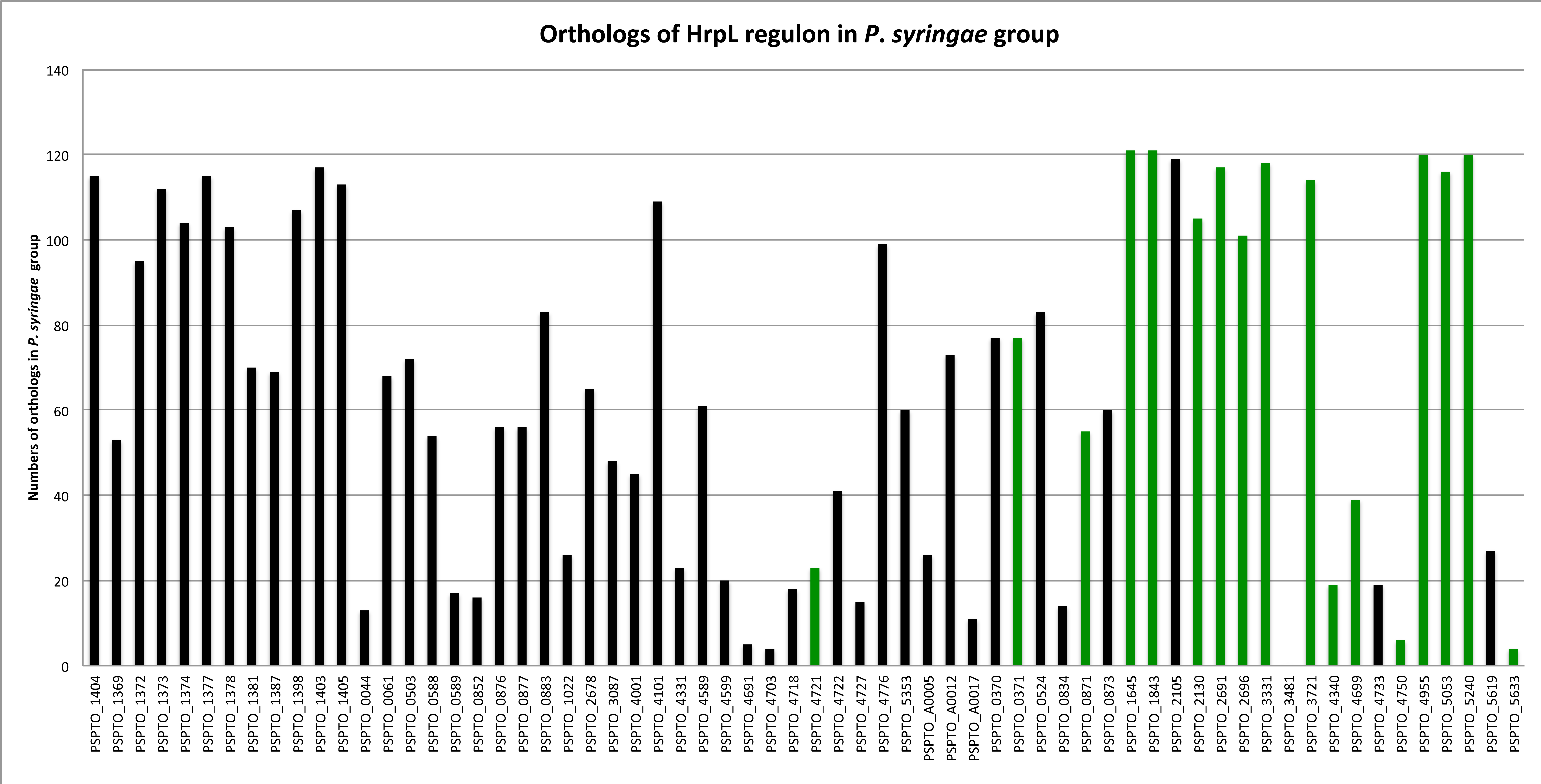

Supplement: Figure S4 — Ortholog inventory of HrpL regulon in P . syringae group. Green represents newly found members; black represents previously annotated regulon members. The values shown represent counts of orthologs of HrpL regulon members across 121 species. (TIF) [file pone.0106115.s004.tif]

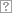

Supplement: Dataset S7 — MEME analysis of sequences upstream from captured 5′-ends using RNA-Seq data from hrpL-FLAG cells at t = 1.5 hours. (ZIP) [file pone.0106115.s016.zip › Dataset S7/help.gif]
