# Supplementary material for: Global Analysis of the HrpL Regulon in the Plant Pathogen Pseudomonas syringae pv. tomato DC3000 Reveals New Regulon Members with Diverse Functions
Source: PLoS One. 2014 Aug 29;9(8):e106115. doi: 10.1371/journal.pone.0106115 (PMC4149516; doi:10.1371/journal.pone.0106115)
Supplement: Table S2 — hrp promoter motif sequences included in the HMM training set. (DOCX) [file pone.0106115.s006.docx]

**Table S2: *hrp* promoter motif sequences included in the HMM training set**

>61504:61536 REVERSE

GGAACTGCAAGCTGGTGCGGCTCCACTAA

>82447:82478 FORWARD

GGAACTCATCACCGCGAATCG-CCACTCA

>404752:404784 FORWARD

GGAACTGCAACGTTGTTTCGG-TCACTCA

>522444:522475 REVERSE

GGAACCGAATCCATATTTCGA-CCACCCA

>550602:550634 REVERSE

GGAACCTGATGCTGCTCAGTGACCACTCA

>572473:572504 FORWARD

GGAACCCATAGAGCCTGCCTG-CCACTTA

>648424:648456 REVERSE

GGAACCGAATCCATCTCGAGGGCCACTCA

>649735:649766 REVERSE

GGAACTGAACCGCTTATGAAA-CCACTCA

>905339:905371 FORWARD

GGAACCAAAACTGGAAAAACATCCACTCA

>921879:921911 REVERSE

GGAACCTCACGCTTAGTGATGACCACGCA

>939413:939445 REVERSE

GGACCCGAATCCGTCTTAAACACCACTCA

>941100:941132 FORWARD

GGAACCGATAACGACTTTTTGGCCACTTA

>946154:946185 FORWARD

GGAACCCAAGAGCCCTTGCGA-CCACACA

>949826:949858 REVERSE

GAAACCGAAACGGCGTTGCTTGCCACACA

>954203:954234 FORWARD

GGAACCGATCCGGTTGCCTGG-CCACTCA

>981177:981209 FORWARD

GGAACCCGATGACACAAGGCG-ACACTCA

>1116378:1116410 REVERSE

GGAACCACATCATGGGTAAAAGCCACGAA

>1504886:1504917 FORWARD

GGAACCGCATCACGTCTTGAA-CCACAGA

>1505219:1505251 FORWARD

GGAACTGTTGTTGCGCAGTGT-GCACTCA

>1507652:1507684 FORWARD

GGAACCGTCAACCGATCCGGGACCACACA

>1510785:1510816 REVERSE

GGAACCGGTCGCTGCGCTTTG-CCACTCA

>1510881:1510913 FORWARD

GGAACTGAAATGCCTATGCCTGCGACTCA

>1519570:1519601 REVERSE

GGAACCCGCTGGCATTGCATG-CCACTCA

>1519666:1519697 FORWARD

GGAACCGTAACGGCGAGCGTG-CCACGTA

>1524204:1524236 FORWARD

GGAACCGATTCGCAGGCTGCTGCCACCTA

>1528184:1528216 FORWARD

GGAACCGCTCGGCGGGTTTGCTCCACTCA

>1536874:1536905 REVERSE

GGAACTGAAATCGATGCTCGA-CCACTTA

>1542621:1542653 REVERSE

GGAACTGATCCGGGACCGTGACCCACTCA

>1543416:1543447 FORWARD

GGAACCAACTTGCACCTTCAA-CCACACA

>1548389:1548420 FORWARD

GGAACCGATTTCGATGAGTCG-CCACACA

>1731421:1731453 REVERSE

GGAACCACTGAAGAGTTTTAAGCCACTCA

>2279883:2279915 FORWARD

GGAACCCTGCGCAGGTCATTGACCACTCA

>2973825:2973856 FORWARD

GGAACCGAGTCACTCA-GTGAACCACTCA

>3470185:3470217 REVERSE

GGAACTCTTTCCTGCTCTTTTGCCACACA

>4515296:4515328 REVERSE

GGAACCGATCCGCTCCCTATGACCACTCA

>4621129:4621160 FORWARD

GGAACTCTTTCCCTGCGCTTT-CCACTCA

>4881097:4881129 FORWARD

GGAACCGAATCCGCCTCAAAGTCCACACA

>5186123:5186154 REVERSE

GGAACCCCTGCGCGTCCAGCG-CCACTCA

>5192613:5192644 REVERSE

GGAACCGGACGAGGCTTTTTA-CCACTCA

>5305220:5305252 FORWARD

GGAACCTTTTTCTTGCATAGCGCCACAGA

>5330688:5330720 FORWARD

GGAACTGCAAGCTGGTGCGGCTCCACTAA

>5344375:5344407 REVERSE

GGAACCGTTAACGGCCCAGCGACTACACA

>5348578:5348610 REVERSE

GGAACCCCACAAGCACTTATGACCACGCA

>5350034:5350065 FORWARD

GGAACCCACGAGCCCTTGTGA-CCACATA

>5355224:5355256 REVERSE

GGAACTCTACGCTTGGCGATGACCACGTA

>5355530:5355562 FORWARD

GGAACTATCTTCCCACACGGAGCCACTTA

>5361707:5361739 REVERSE

GGAACCAAATCCGCCTCAAAGTCCACTCA

>5418197:5418228 REVERSE

GGAACCAGATCTCGTTGCTTG-CCACCAA

>6085756:6085788 FORWARD

GGAACCGCCTCGAGCAGAGGCTCCACTCA

>15821:15849 FORWARD

GGAACTGCCGGCTGGTGCGGCTCCACTAA

>406210:406238 FORWARD

GGAACCGATCTCGCCATCGAGGTAACTCA

>880830:880858 FORWARD

GGAACCACCTATTTCACGTCGACAACCAA

>922925:922953 REVERSE

GGAACCAAATTGCTGGCTACGCTAACCAA

>939675:939703 FORWARD

GGAACCCATCAGCACAAGCGCACCACGAA

>1115513:1115541 REVERSE

GGAACTAAATAATTTCCTAGG-CGACTAA

>1802305:1802333 FORWARD

GGAACTGCGCCGCGACCTCAAACCACAAA

>2012108:2012136 FORWARD

GCAACCAAACTGAACCATTCGGCCACTCA

>2304331:2304359 REVERSE

GGAACTGAGTGACGGAAACGTTTCACTAA

>2984435:2984463 FORWARD

GGAACTTTCGGTACAGGAGCTACCACAAA

>2990249:2990277 REVERSE

GGAACTGTTTTCTGGCAATGGCCCACTGA

>3768950:3768978 REVERSE

GGAACTGTTGGTCAAGCGTTTGCCACTGA

>3929005:3929032 FORWARD

GGAACT-GATTCCCAGGATCGGTGACTAA

>4199604:4199632 FORWARD

GGAACTGGTCGGCATCCCTGAGCCACATA

>4457004:4457031 REVERSE

GGAAG-GACATCCTCTTCGTGATCACCTG

>4895201:4895228 REVERSE

GGAACT-TCAAGGCTGACGCGGTAACTCA

>5328022:5328050 REVERSE

GGAACCGATAACAAATTTTTGGCCACATA

>5346761:5346788 REVERSE

GGAAC-GTGTCCGCTGTTACGGCCACTTA

>5384480:5384507 FORWARD

GGAACC-CATGGAGCTTGTTAGCCACATA

>5616671:5616699 FORWARD

GGAACCATTGAGCCTGTTCGCGCCACTGA

>5751475:5751504 FORWARD

GGAAACCGGACAGCAGTTGCTGGCCACTC

>5960164:5960192 REVERSE

GGATCAAAAATGGGCCTGGATGCCACTAA
