# Supplementary material for: Global Analysis of the HrpL Regulon in the Plant Pathogen Pseudomonas syringae pv. tomato DC3000 Reveals New Regulon Members with Diverse Functions
Source: PLoS One. 2014 Aug 29;9(8):e106115. doi: 10.1371/journal.pone.0106115 (PMC4149516; doi:10.1371/journal.pone.0106115)
Supplement: Table S4 — Additional hrp promoter-like sequences with limited experimental support. (DOCX) [file pone.0106115.s008.docx]

**Table S4: Additional *hrp* promoter-like sequences with limited experimental support**

| Operon | Function | *hrp* promoter like sequence | *hrp* promoter E-value | coordinate | ChIP-Seq | RNA-Seq | gfp/od  on KB | gfp/od  on hmm |
| --- | --- | --- | --- | --- | --- | --- | --- | --- |
| 0816 | type IV pilus biogenesis protein | GGAACCACCTATTTCACGTCGACAACCAA | 0.0031 | 880830..880858 | weak | low | 16.6 ± 18.4 | 180.3 ± 28.7 |
| 1654* | conserved protein of unknown function | GAAACCGCAAGATCAAACGGCACCACCCA | 0.0055 | 1819661..1819689 | weak | x | ND | ND |
| 3019 | oxidoreductase, Gfo/Idh/MocA family | GGAAC-GCGACTATGCTGCGGGCCACTCA | 0.0024 | 3393411..3393438 | weak | low | ND | ND |
| 3122 | 6-phosphogluconate dehydrogenase | GGAACCGACGTTCTGCCTCGCTTGACTAA | 0.0072 | 3508787..3508815 | weak | low | ND | ND |
| 4580 | conserved protein of unknown function | GGAACTGGTGCAGGGCGCTT-GCCACGAA | 0.0013 | 5176134..5176161 | weak | low | ND | ND |

Operon: PSPTO identifier for operon/gene immediately downstream from *hrp* promoter like sequence.

*1654: predicted *hrp* promoter is antisense relative to this gene.

Function: annotated function for identifying gene.

Sequence: potential –35 and -10 regions are underlined and aligned

*hrp* promoter E-value: E-value for match to profile HMM.

Coordinate: DC3000 genome coordinate for *hrp* promoter-like sequence

ChIP-Seq: qualitative assessment of HrpL-binding activity.

RNA-Seq: “low” for low abundance transcripts (less than 100 reads in the coding region), “x” for no reads.

GFP/OD: reporter fluorescence normalized to OD_600_ for reporter constructs cultured in KB or *hrp-* minimal media. ND indicates not determined.
