# Supplementary material for: Global Analysis of the HrpL Regulon in the Plant Pathogen Pseudomonas syringae pv. tomato DC3000 Reveals New Regulon Members with Diverse Functions
Source: PLoS One. 2014 Aug 29;9(8):e106115. doi: 10.1371/journal.pone.0106115 (PMC4149516; doi:10.1371/journal.pone.0106115)
Supplement: Table S5 — Data for all hrp promoters. (DOCX) [file pone.0106115.s009.docx]

**Table S5: Data for all *hrp* promoters**

| **Operon** | **Function** | ***hrp* promoter coordinate** | **Evidence** | **Distance to TSS^a^** | **TSS^b^ Counts** | **Published data** | | |
| --- | --- | --- | --- | --- | --- | --- | --- | --- |
|  |  |  |  |  |  | **Distance to TSS^c^** | **TSS^d^ Counts** | **Promoters and genes** |
| 44 | Type III effector HopK1 | 61504..61536 | HBQS | 51, 81 | 4, 4634 | 1, 8 | 1, 47 | CM* 13.2 |
| 61 | Type III effector HopY1 | 82447..82478 | HBQS | 4, 6 | 101, 597 | 4, 5 | 99, 965 | FCM 16.3 Y |
| 370 | MATE efflux family protein | 404752..404784 | HBS | 3, 6 | 4, 2725 | 6, 6 | 390, 390 | FCM_0_ 13.2 Y |
| 371 | *iaaL* indoleacetate-lysine ligase | 406210..406238 | HBIQS | 2, 6 | 4, 9483 | 2, 6 | 1, 2452 | FCM_0_ 10 Y |
| 503-(502-501) | Type III chaperone protein ShcF-(hopF2-hopU1) | 550602..550634 | HBQS | 77, 79 | 4, 42 | 6, 6 | 67, 67 | M-FCM_0_-M_0_ 18.8 Y |
| 524 | M20/M25/M40 family peptidase | 572473..572504 | HBS | 4, 7 | 4, 509 | 5, 7 | 22, 34 | M* 18.3 Y |
| 588 | Type III effector HopH1 | 648424..648456 | HBQS | 22, 80 | 4, 736 | 6, 7 | 22, 598 | CM 26.1 Y |
| 589 | Type III effector HopC1 | 649735..649766 | HBQS | 60, 80 | 4, 1220 | 7, 7 | 120, 120 | FCM 20.4 Y |
| 834-(835-836-837-838) | alcohol dehydrogenase, zinc-containing-(ribD C-terminal domain protein-conserved domain protein-conserved protein of unknown function-major facilitator family transporter) | 905339..905371 | HBQS | 2, 6 | 4, 3621 | 4, 6 | 11, 115 | CM*-CM_0_-CM_0_-M_0_- M_0_ 19.2 Y |
| 852 | Type III effector HopAJ1 | 921879..921911 | HBQS | 42, 81 | 8, 1518 | 6, 8 | 4, 762 | FCM 15.5 Y |
| 871 | macrolide efflux protein, putative | 939675..939703 | HBIQS | 4, 6 | 122, 643 | 4, 6 | 17, 51 | Y |
| 873 | amidinotransferase family protein | 941100..941132 | HBQS | 2, 4 | 147, 774 | 2, 6 | 32, 294 | M_0_ 19.2 Y |
| 876 | Type III effector HopD1 | 946154..946185 | HBQS | 4, 5 | 4, 206 | 2, 5 | 1, 75 | FCM 20.4 Y |
| 877 | Type III effector HopQ1-1 | 949826..949858 | HBQS | 5, 80 | 17, 6590 | 5, 7 | 1, 559 | CM 23.3 Y |
| 883 | Type III effector HopR1 | 954203..954234 | HBQS | 4, 7 | 29, 833 | 2, 7 | 1, 2361 | FCM 20.7 Y |
| 1022 | Type III effector HopAM1-1 | 1116378..1116410 | HBQS | 43, 79 | 4, 6838 | x | x | FCM 18.4 |
| 1369 | Type III chaperone protein ShcN | 1504886..1504917 | HBQS | 2, 5 | 164, 2826 | 2, 5 | 10, 205 | FM 18.4 Y |
| 1370 | Type III effector HopN1 | 1505219..1505251 | QS | 5, 6 | 42, 307 | 0, 6 | 5, 34 | FCM 12.8 Y |
| 1372 | Type III effector HopAA1-1 | 1507652..1507684 | HBQS | 3, 3 | 42, 42 | 3, 3 | 7, 7 | FCM 18.7 Y |
| 1373 | Type III helper protein HrpW1 | 1510785..1510816 | HBQS | 77, 80 | 4, 307 | 3, 7 | 3, 670 | FCM 15.4 Y |
| 1374-(1375-1376) | type III chaperone ShcM-(hopM1-shcE) | 1510881..1510913 | HBQS | 3, 6 | 4, 458 | 4, 6 | 54, 114 | CM 17.9 Y |
| 1377 | Type III effector protein AvrE1 | 1519570..1519601 | HBQS | 67, 80 | 4, 967 | 6, 7 | 1, 238 | CM_0_ 15.2 Y |
| 1378 | membrane-bound lytic murein transglycosylase D | 1519666..1519697 | HBQS | 4, 6 | 50, 122 | 4, 6 | 45, 67 | FCM 20.7 Y |
| 1381-(1382-1383-1384-1385-1386) | Type III helper protein HrpA1-(hrpz1-hrpB-hrcJ-hrpD-hrpE) | 1524204..1524236 | HBQS | 3, 6 | 29, 58959 | 3, 6 | 2, 3307 | CM-CM-M-M-M-M 20.6 Y |
| 1387-(1388-1389-1390-1391) | Type III secretion protein HrpF-(hrpG-hrcC-hrpT-hrpV) | 1528184..1528216 | HBQS | 5, 6 | 21, 1350 | 5, 6 | 6, 654 | CM_1_-M-M- M_1_-M 20.4 Y |
| 1398-(1397-1396-1395-1394-1393-1392) | Type III secretion protein HrpP-(hrcQa-hrcQb-hrcR-hrcS-hrcT-hrcU) | 1536874..1536905 | HBQS | 72, 80 | 4, 341 | 5, 6 | 12, 255 | M-M-M-M- M_1_- M_0_-M_0_ 20.4 Y |
| 1403-(1402-1401-1400-1399) | Type III secretion protein HrpJ-(hrcV-hrpQ-hrcN-hrpO) | 1542621..1542653 | HBQS | 19, 80 | 4, 282 | 5, 7 | 2, 60 | CM-M-M-M_0_-M 20.4 Y |
| 1405-(1406) | Type III helper protein HrpK1-(HopB1) | 1543416..1543447 | HBQS | 6, 6 | 151, 151 | 5, 6 | 2, 388 | CM 16.4 Y |
| 1645 | MarR family transcriptional regulator | 1802305..1802333 | HBIQS | 5, 7 | 269, 408 | 3, 5 | 1, 41 | M* 12.3 |
| 1843 | aspartate kinase | 2012108..2012136 | HBIQS | 5, 6 | 168, 1707 | 0, 6 | 1, 116 | Y |
| 2105 | thiamine biosynthesis lipoprotein, putative | 2279883..2279915 | HBS | 1, 6 | 147, 7023 | 1, 6 | 1, 91 | FCM* 22.8 Y |
| 2130 | LuxR family DNA-binding response regulator | c(2304331..2304359) | HBIQS | 79, 80 | 21, 185 | 7, 7 | 32, 32 | M_0_ 11.3 Y |
| 2678-(2679) | Type III helper protein HopP1-(protein of unknown function) | 2973825..2973856 | HBQS | 0, 3 | 21, 400 | 1, 3 | 4, 25 | FCM 19.6 Y |
| 2691 | TerC family membrane protein | 2984435..2984463 | HBIQS | 5, 5 | 67, 67 | 4, 5 | 1, 23 | M* Y |
| 2696 | mutT/nudix family protein | c(2990249..2990277) | HBIQS | 60, 79 | 4, 980 | 6, 17 | 31, 160 | M_0_ 11.6 |
| 3087 | Type III effector HopAB2 | 3470185..3470217 | HBS | 37, 79 | 4, 4735 | 6, 6 | 908, 908 | FCM 21.2 Y |
| 3331 | protease inhibitor Inh | c(3768950..3768978) | HBIQS | 78, 80 | 8, 59 | 0, 22 | 1, 709 |  |
| 3481 | Hypothetical protein | 3929005..3929032 | HBQS | 4, 5 | 17, 841 | 2, 5 | 1, 810 |  |
| 3489-(3488) | sugar ABC transporter, ATP-binding protein-(sugar ABC transporter, permease protein) |  |  | x | x | x | x | M-M 11.6 |
| 3721 | fabI enoyl-(acyl-carrier-protein) reductase | 4199604..4199632 | HBIS | 6, 6 | 84, 84 | 1, 6 | 1, 3 | M_0_ 11 |
| 3948_49 | Hypothetical protein | c(4457004..4457031) | HBIQS | 30, 30 | 8, 8 | 28, 70 | 1, 453 |  |
| 4001 | Type III effector protein AvrPto1 | 4515296..4515328 | HBQS | 27, 80 | 4, 711 | 4, 7 | 4, 1194 | FCM 22.3 Y |
| 4101 | Type III effector HopAK1 | 4621129..4621160 | HBQS | 1, 7 | 4, 147 | 5, 7 | 22, 253 | FCM 15.1 |
| 4331 | Type III effector HopE1 | 4881097..4881129 | HBQS | 4, 7 | 4, 4088 | 5, 7 | 12, 348 | CM 23.6 Y |
| 4340 | insecticidal toxin protein, putative | c(4895201..4895228) | HBIQS | 80, 80 | 25, 25 | 5, 7 | 1, 3 |  |
| 4589-(4588) | Type III chaperone ShcS2-(hopS2) | 5186123..5186154 | HBQS | 30, 79 | 4, 2031 | 5, 6 | 1, 223 | CM_0_-CM_1_ 18.7 Y |
| 4599-(4597) | Type III chaperone ShcS1-(hopS1) | 5192613..5192644 | HBQS | 43, 80 | 4, 892 | 2, 7 | 1, 1277 | FM-CM 24.2 Y |
| 4691 | Type III effector HopAD1 | 5305220..5305252 | HBS | 3, 5 | 4, 971 | 4, 5 | 3, 55 | CM_0_ 8.7 Y |
| 4699 | non-ribosomal peptide synthetase, terminal | c(5328022..5328050) | HBIQS | 19, 77 | 4, 235 | 2, 4 | 9, 25 | M_0_ 16.8 |
| 4703-(4704-4705) | Type III effector HopAQ1-(corR-corS) | 5330688..5330720 | HBS | 3, 7 | 50, 122 | 1, 7 | 1, 16 | CM_1_ 13.2 Y |
| 4718 | Type III effector HopAA1-2 | 5344375..5344407 | HBQS | 36, 81 | 4, 3427 | 1, 8 | 1, 493 | M_*_ 18.8 Y |
| 4721 | Type III chaperone ShcV | c(5346761..5346788) | HBIQS | 41, 79 | 4, 261 | 3, 74 | 1, 25 | M_0_ 12.2 Y |
| 4722 | Type III effector HopAO1 | 5348578..5348610 | HBQS | 1, 79 | 8, 38 | 5, 6 | 2, 17 | FCM_0_ 13.8 Y |
| 4724-(4725) | Type III effector HopD-(IS52, transposase) | 5350034..5350065 | HBQS | 4, 5 | 4, 67 | 4, 61 | 8, 410 | M_1_ 16 |
| 4727 | Type III effector HopG1 | 5355224..5355256 | HBQS | 74, 81 | 4, 63 | 6, 8 | 1, 10 | CM 14.4 Y |
| 4733 | Hypothetical protein | 5361707..5361739 | HBS | 60, 79 | 4, 400 | 6, 8 | 174, 239 | M* 22.2 Y |
| 4750 | Hypothetical protein | 5384480..5384507 | HBIQS | 4, 6 | 59, 261 | 4, 6 | 3, 28 |  |
| 4776 | Type III effector HopI1 | 541819.. 5418228 | HBQS | 76, 80 | 4, 908 | 5, 7 | 13, 128 | FCR 16.5 Y |
| 4955 | bifunctional thiosulfate | 5616671..5616699 | HBIQS | 3, 7 | 412, 421 | 0, 3 | 6, 254 | M_0_ 10.3 |
| 5053 | Hypothetical protein | 5751475..5751504 | HBIQS | 4, 5 | 4, 1156 | 4, 5 | 1, 84 | M_0_ 19.8 |
| 5240 | CDP-6-deoxy-delta-3,4-glucoseen reductase | c(5960164..5960192) | HBIQS | 0, 80 | 4, 248 | 0, 6 | 1, 90 |  |
| 5353-(5354) | Type III chaperone protein ShcA-(hopA1) | 6085756..6085788 | HBQS | 1, 2 | 71, 172 | 0, 2 | 1, 328 | CM-FCM 17.5 |
| 5616-(0474-0473) | Hypothetical protein-(HopAS1 (interrruption-N)-HopAS1 (interruption-C)) | 522444..522475 | HBS | 75, 79 | 4, 34 | 2, 6 | 77, 876 | FM* 14.7 |
| 5617 | conserved Hypothetical gene | 939413..939445 | HB*S | 76, 77 | 13, 67 | 3, 45 | 2, 107 | M* 20.2 |
| 5618 | Type III effector pseudogene hopAT1 | c(922925..922953) | HIQS | 29, 79 | 4, 214 | 6, 6 | 37, 37 | M_1_ <6.0 |
| 5619-(0901) | PSPTO_5619-(hopAG1) | 981177..981209 | HBS | 6, 6 | 122, 122 | x | 0, 0 | CM_0_ 11.7 Y |
| 5620-(1568) | PSPTO_5620-(hopAF1) | 1731421..1731453 | HBS | 75, 77 | 8, 114 | 5, 5 | 4, 4 | M_0_-CM* 17 Y |
| 5622 | PSPTO_5622 | 1548389..1548420 | HBS | 2, 5 | 8, 20265 | 3, 5 | 1, 1512 | M_0_ 16.1 Y |
| 5623 | PSPTO_5623 | 5355530..5355562 | HBS | 3, 5 | 50, 1939 | 5, 5 | 6, 6 | M 10.9 |
| 5633 | conserved protein of unknown function | 15821..15849 | HBIQS | 1, 7 | 8, 31927 | x | 0, 0 |  |
| A0005 | type III effector HopAM1-2 | c(6595..6627) | HBS | 41, 77 | 134, 218196 | x | x | FM 18.4 |
| A0012 | type III effector HopX1 | c(16103..16135) | HBS | 31, 78 | 134, 18250 | x | x | FM 18.1 |
| A0017-(A0018-A0019) | type III chaperone ShcO1-(hopO1-1-HopT1-1) | 19658..19690 | HBS | 2, 4 | 134, 61326 | x | x | M 16.4 |
| B0003 | (identical to PSPTO_5633) |  |  |  |  |  |  |  |
| B0078 | hypothetical protein | c(6894..6926) | HBS | 32, 79 | 49, 166407 | x | x | M_2_ 16.0 |

Operon: PSPTO identifier for genes immediately downstream from promoter. Identifiers in parentheses indicate that the operon contains multiple genes. Operons are adapted from Table 2 in Ferreira *et al.* [[23](#_ENREF_23)]

Function: annotated function for operon-identifying genes.

Coordinate: DC3000 genome coordinate for the region bracketing the –35 and –10 regions of the promoter. “c” designates that the promoter is found on the complementary strand.

Evidence: Experimental evidence for *hrp* promoters from this study.

H: *hrp* promoter motif found;

B: Binding activity for HrpL (ChIP-qPCR and/or ChIP-Seq) observed;

(*) ChIP-Seq enrichment occurs between two divergent promoters. The asterisk designates the promoter located further from the enriched area.

I: Induction observed in promoter fusion (threshold = 2.4; 2 x negative control);

Q: HrpL dependent transcription of regions downstream from *hrp* promoters in DC3000 in qRT-PCR.

S: mRNA 5’-end captured (TSS). Absolute values for read counts appear in ^b^ and ^d^.

^a^ Distance between *hrp* promoters (3’-end of –10 region) and captured 5’-end within 100 bps using data from this study. The first value is the distance to the closest captured end. The second value is the distance to the captured end with the most sequence read counts within 100 bps from the *hrp* promoter.

^b^ Read counts from 5’-end capture data within 100bps using data from this study. The first value is read count for the closest captured 5’-end to the *hrp* promoter. The second value is read count for the captured end with the most sequence reads within 100 bps from the *hrp* promoter. Values are based on one replicate as described in the text and methods.

Published data:

^c^ Distance between *hrp* promoter (5’-end of –10 region) and captured 5’-end within 100 bps using data from Filiatrault *et al.* [[46](#_ENREF_46)]. The first value is the distance to the closest captured 5’-end. The second value is the distance to the captured end with the most sequence read counts within 100 bps from the *hrp* promoter.

^d^ Read counts for captured 5’-ends within 100 bps using data from Filiatrault *et al.* [[46](#_ENREF_46)]. The first value is the read count for the closest captured 5’-end to the *hrp* promoter. The second value is the read count for the captured end with the most sequence read counts within 100 bps from the *hrp* promoter.

• Promoters and genes: promoters and differentially expressed genes associated with the HrpL regulon reported elsewhere.

- Y: Mucyn *et al*. [[26](#_ENREF_26)]
- C: Chang *et al*. [[22](#_ENREF_22)]
- F: Fouts *et al*. [[21](#_ENREF_21)]
- M: Ferreira *et al.* [[23](#_ENREF_23)]exhibited differential expression in microarray (M); exhibited differential expression in microarray and/or RT-PCR but did not pass other criteria (M*); genes present on microarray but no evidence for differential expression (M_o_); genes not on microarray and not tested by RT-PCR (M_1_); evidence for existence of peptide fragment detected by mass spectrometry (M_2_). The score for the match between the hidden Markov model and the *hrp* promoter reported by Ferreira *et al.*

Background colors:

BLUE: experimental evidence for HrpL-dependent expression as reported in [[23](#_ENREF_23)], and [[21](#_ENREF_21), [22](#_ENREF_22)]. The first study used microarray to compare a *hrpL* deletion with wild-type DC3000 in *hrp* minimal medium. The second and third studies compare a *hrpL* deletion to a strain in which HrpL was overexpressed.

GREEN: experimental evidence reported in [[23](#_ENREF_23)], but not in [[21](#_ENREF_21), [22](#_ENREF_22)].

ORANGE: experimental evidence reported in [[21](#_ENREF_21), [22](#_ENREF_22)], but not in [[23](#_ENREF_23)].

WHITE: experimental evidence for HrpL-dependent expression only in a recent RNA-Seq by Mucyn *et al*. [[26](#_ENREF_26)].

GRAY: no previously experimental evidence for HrpL-dependent expression.
